# CD147‐K148me2‐Driven Tumor Cell‐Macrophage Crosstalk Provokes NSCLC Immunosuppression via the CCL5/CCR5 Axis

**DOI:** 10.1002/advs.202400611

**Published:** 2024-06-14

**Authors:** Ke Wang, Xiaohong Chen, Peng Lin, Jiao Wu, Qiang Huang, Zhi‐Nan Chen, Jiale Tian, Hao Wang, Ye Tian, Mingyan Shi, Meirui Qian, Bengang Hui, Yumeng Zhu, Ling Li, Rui Yao, Huijie Bian, Ping Zhu, Ruo Chen, Liang Chen

**Affiliations:** ^1^ Department of Cell Biology of National Translational Science Center for Molecular Medicine and Department of Clinical Immunology of Xijing Hospital Fourth Military Medical University Xi'an 710032 China; ^2^ State Key Laboratory of New Targets Discovery and Drug Development for Major Diseases China; ^3^ School of Medicine Shanghai University Shanghai 200444 China; ^4^ Department of Thoracic Surgery of Tangdu Hospital Fourth Military Medical University Xi'an 710038 China

**Keywords:** CCL5, CD147‐K148me2, non‐small cell lung cancer, NSD2, tumor‐associated macrophages

## Abstract

Immunosuppression is a major hallmark of tumor progression in non‐small cell lung cancer (NSCLC). Cluster of differentiation 147 (CD147), an important pro‐tumorigenic factor, is closely linked to NSCLC immunosuppression. However, the role of CD147 di‐methylation in the immunosuppressive tumor microenvironment (TME) remains unclear. Here, di‐methylation of CD147 at Lys148 (CD147‐K148me2) is identified as a common post‐translational modification (PTM) in NSCLC that is significantly associated with unsatisfying survival outcomes among NSCLC sufferers, especially those in the advanced stages of the disease. The methyltransferase NSD2 catalyzes CD147 to generate CD147‐K148me2. Further analysis demonstrates that CD147‐K148me2 reestablishes the immunosuppressive TME and promotes NSCLC progression. Mechanistically, this modification promotes the interaction between cyclophilin A (CyPA) and CD147, and in turn, increases CCL5 gene transcription by activating p38‐ZBTB32 signaling, leading to increased NSCLC cell‐derived CCL5 secretion. Subsequently, CD147‐K148me2‐mediated CCL5 upregulation facilitates M2‐like tumor‐associated macrophage (TAM) infiltration in NSCLC tissues via CCL5/CCR5 axis‐dependent intercellular crosstalk between tumor cells and macrophages, which is inhibited by blocking CD147‐K148me2 with the targeted antibody 12C8. Overall, this study reveals the role of CD147‐K148me2‐driven intercellular crosstalk in the development of NSCLC immunosuppression, and provides a potential interventional strategy for PTM‐targeted NSCLC therapy.

## Graphical abstract

Di‐methylation of CD147 at Lys148 (CD147‐K148me2) mediated by the methyltransferase NSD2 is a common post‐translational modification in non‐small cell lung cancer (NSCLC), which values significantly in unsatisfactory survival outcomes among NSCLC sufferers. This modification promotes the CyPA‐CD147 interaction and in turn increases NSCLC cell‐derived CCL5 secretion by activating p38‐ZBTB32 signaling, which facilitates M2‐tumor‐associated macrophage infiltration in NSCLC tissues via the CCL5/CCR5 axis. Targeting CD147‐K148me2 is a promising therapeutic strategy for NSCLC.

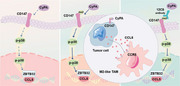

## 1 Introduction

The tumor microenvironment (TME) is a highly intricate environment composed of inflammatory cells, fibroblasts, and the extracellular matrix and is closely linked to complex biological behaviors in tumors.^[^ ^1^ ^]^ Notably, the interactions between tumor cells and immune components, including myeloid‐derived suppressor cells (MDSCs), regulatory T cells (Tregs), and tumor‐associated macrophages (TAMs), contribute to the formation of an immunosuppressive TME, leading to deficient immunosurveillance and unsatisfactory clinical outcomes.^[^ ^2^, ^3^, ^4^, ^5^ ^]^ As the most abundant immune population of the TME, TAMs, which are commonly divided into two phenotypes, namely, anti‐tumorigenic TAMs (M1‐TAMs) and pro‐tumorigenic TAMs (M2‐TAMs), have gained widespread attention in oncogenic fields owing to their diversity and complexity.^[^ ^6^, ^7^, ^8^ ^]^ Unlike M1 phenotype‐mediated anti‐tumor immunity which involves antigen presentation and pro‐inflammatory cytokine secretion, M2‐TAMs are characterized by high expression of inhibitory molecules, such as PD‐L1 and Tim3, which inhibit the activities of cytotoxic T cells and natural killer cells that can eradicate tumors and remodel the immunosuppressive TME during tumor evolution, leading to malignant behaviors.^[^ ^6^, ^9^ ^]^ Although many studies have investigated the carcinogenic effects of M2‐TAMs in multiple cancers, the detailed mechanisms of immunosuppressive TME reconstruction induced by interactions between tumor cells and M2‐TAMs have not yet been fully elucidated.

As a common post‐translational modification (PTM), protein methylation, including histone and non‐histone methylation, has a critical effect on tumor progression by regulating protein‐protein interactions, protein‐DNA interactions, protein subcellular localization, and protein stability^[^ ^10^ ^]^ and is closely associated with M2‐TAM‐mediated immunosuppressive TME remodeling.^[^ ^11^, ^12^ ^]^ For histones, H3K4 tri‐methylation increases tumor cell‐derived CCL2 secretion by activating CCL2 transcription, which promotes M2‐TAM recruitment and activation, leading to lymphatic metastasis in bladder cancer.^[^ ^13^ ^]^ G9A‐mediated H3K9 di‐methylation inhibits Fbxw7 transcription in glioma stem cells and subsequently promotes immune suppression by increasing the infiltration of M2‐TAMs in the TME.^[^ ^14^ ^]^ In addition, non‐histone methylation is involved in M2‐TAM‐mediated immunosuppression. For example, MCT1 tri‐methylation mediated by the methyltransferase SETDB1 sustains MCT1 stabilization and in turn promotes tumor glycolysis and M2‐like polarization of TAMs in colorectal cancer, which contributes to tumor immunosuppression.^[^ ^15^ ^]^ These studies indicate that abnormal methylation exacerbates the immunosuppressive TME by regulating tumor cell‐macrophage intercellular crosstalk, suggesting that targeting methylation or methyltransferases in the TME may be a strategy for tumor treatment.

Consistent with findings from our previous work, many studies have confirmed that the tumor‐associated antigen CD147 is highly expressed in various tumors and promotes tumor progression through various biological processes.^[^ ^16^, ^17^, ^18^, ^19^ ^]^ As the role of CD147 in tumor progression has been further elucidated, the PTMs of CD147 have been extensively investigated owing to their diversity and heterogeneity. Among them, di‐methylation is a relatively common PTM of CD147 and is found at multiple lysine residues. The di‐methylation of CD147‐K234 catalyzed by the methyltransferase KMT5A is highly expressed in non‐small cell lung cancer (NSCLC), which drives tumor glycolysis and lactate export by enhancing the interaction between CD147 and MCT4, and promotes tumor progression.^[^ ^20^ ^]^ However, compared to that in para‐carcinoma tissues, SETDB1‐mediated di‐methylation of CD147‐K71 is substantially decreased in NSCLC, increasing tumor cell sensitivity to chemotherapy‐induced apoptosis.^[^ ^21^ ^]^ These studies show that different di‐methylations of CD147 have distinct biological functions in mediating tumor progression. However, the precise mechanisms by which CD147 di‐methylation drives M2‐TAM‐mediated tumor immunosuppression are still unclear.

In our study, we discovered that methyltransferase NSD2‐mediated CD147 di‐methylation at Lys148 (CD147‐K148me2) was significantly greater in NSCLC tissues than in para‐carcinoma tissues. This targeted modification increased NSCLC cell‐derived CCL5 secretion via CyPA‐CD147‐p38‐ZBTB32 signaling, which promoted NSCLC immunosuppression via CCL5/CCR5 axis‐dependent tumor cell‐macrophage crosstalk. Our study demonstrates the positive effect of CD147 di‐methylation on the formation of an immunosuppressive TME and provides a promising interventional strategy for NSCLC therapeutic regimens by targeting PTMs.

## 2 Results

### 2.1 CD147 Di‐Methylation at Lys148 Is Increased in NSCLC Patients

CD147 is a transmembrane protein that plays a crucial role in tumor progression. The biological function of CD147 mainly depends on its extracellular domain (ECD). Interactions between ligands or extracellular signals and CD147‐ECD initiate a series of intracellular biological chemical reactions and subsequently trigger signal transduction. A previous study identified a panel of lysine residues of CD147‐ECD modified by di‐methylation in NSCLC samples.^[^ ^20^ ^]^ To screen for differential di‐methylation of CD147‐ECD between NSCLC and para‐carcinoma tissues, we collected 20 pairs of NSCLC and para‐carcinoma tissues and measured the abundance of CD147 di‐methylation at the residues of the CD147‐ECD using liquid chromatography‐tandem mass spectrometry (LC‐MS/MS). The di‐methylation of CD147 at Lys75, 111, 127, and 148 was significantly greater in the NSCLC tissues than in the para‐carcinoma tissues, while the di‐methylation of CD147 at Lys71 was lower in the NSCLC tissues than in the para‐carcinoma tissues (Figure S1A, Supporting Information). The ratio of the modified peptide‐spectrum matches (PSMs) to unmodified PSMs for the peptide K148 was the highest among all the di‐methylated lysine residues (Figure S1B, Supporting Information). Hence, we chose CD147‐K148me2 for further investigation (Figure S1C,D, Supporting Information).

**Figure 1:**
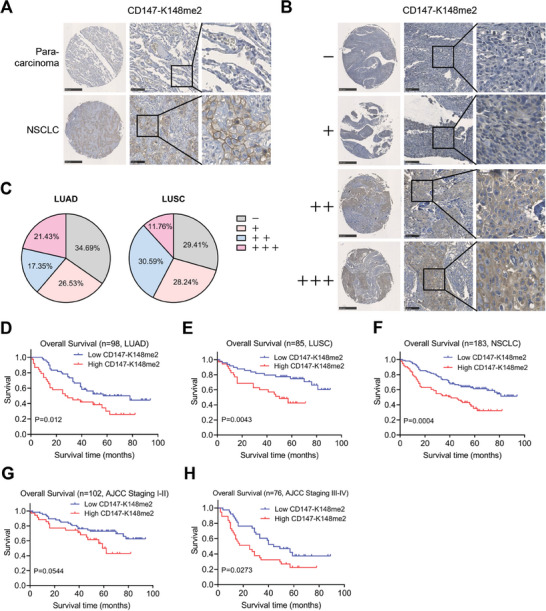
CD147‐K148me2 expression is closely associated with the prognosis of NSCLC patients. A) The expression of CD147‐K148me2 in NSCLC tissues and their corresponding para‐carcinoma tissues, scale bar, 500 µm (left) and 100 µm (middle). B) Different levels (–, +, ++, and +++) of CD147‐K148me2 expression in NSCLC tissues, scale bar, 500 µm (left) and 100 µm (middle). C) The rate of CD147‐K148me2 expression in LUAD and LUSC. D–F) Overall survival of individuals with high and low CD147‐K148me2 expression in the LUAD (P = 0.012) (D), LUSC (P = 0.0043) (E), and NSCLC (P = 0.0004) (F) cohorts. G,H) Overall survival of NSCLC patients with high and low CD147‐K148me2 expression in the early NSCLC (P = 0.0544) (G) and advanced NSCLC (P = 0.0273) (H) cohorts.

To investigate the expression of CD147‐K148me2 in NSCLC samples, we developed a monoclonal antibody (12C8) specifically targeting CD147‐K148me2 by immunizing mice with the CD147‐K148me2 peptide (Figure S2A, Supporting Information). The subtype of the 12C8 antibody was IgG1 (Figure S2B, Supporting Information). Dot blot and enzyme‐linked immunosorbent assays (ELISAs) revealed the strong specificity of 12C8 against CD147‐K148me2; this antibody exclusively binds to the peptide CD147‐K148me2 without cross‐reactivity with the peptide CD147‐K148 or peptides containing other di‐methylated lysine residues of CD147 (Figure S2C–E, Supporting Information). Then, 12C8 was used to determine the CD147‐K148me2 levels in NSCLC and para‐carcinoma tissues by immunohistochemical (IHC) staining, and CD147 itself was also assessed as a reference. According to the expression levels of CD147‐K148me2 and CD147, the NSCLC tissues were divided into four groups, namely, negative (–), weak (+), moderate (++), strong (+++). The percentages of CD147‐K148me2‐positive tissues were 65% in lung adenocarcinoma (LUAD) and 71% in lung squamous cell carcinoma (LUSC); these values were slightly lower than those of CD147, and both molecules were barely detectable in para‐carcinoma tissues (**Figure** **1**A–C; Figure S3A–C, Supporting Information). Then, negative (–) and weak (+) samples were classified as the low group, while moderate (++) and strong (+++) samples were classified as the high group. Clinical analysis revealed that the expression of CD147‐K148me2 was closely linked to gender, tumor size, and pathological staging in LUAD patients (Table S1, Supporting Information), while no significant correlation was found in LUSC patients (Table S2, Supporting Information). Moreover, the expression of CD147 was associated with tumor size and N staging in LUSC patients, while no correlation was found in LUAD patients (Tables S3,4, Supporting Information). Kaplan‐Meier analysis revealed that high expression of both CD147‐K148me2 and CD147 was significantly positively associated with poor overall survival in NSCLC patients (Figure 1D–F; Figure S3D–F, Supporting Information). For patients with NSCLC in the earlier stages (AJCC stages I‐II), a high CD147 level was associated with unsatisfactory clinical outcomes, whereas a high CD147‐K148me2 level worked for NSCLC patients in the advanced stages (AJCC stages III‐IV) (Figure 1G,H; Figure S3G,H, Supporting Information). These findings reveal that CD147‐K148me2 is highly expressed in NSCLC and has prognostic value for NSCLC patients, especially those in the advanced stages of the disease.

### 2.2 The Methyltransferase NSD2 Acts on Lys148 of CD147 to Generate CD147‐K148me2

**Figure 2:**
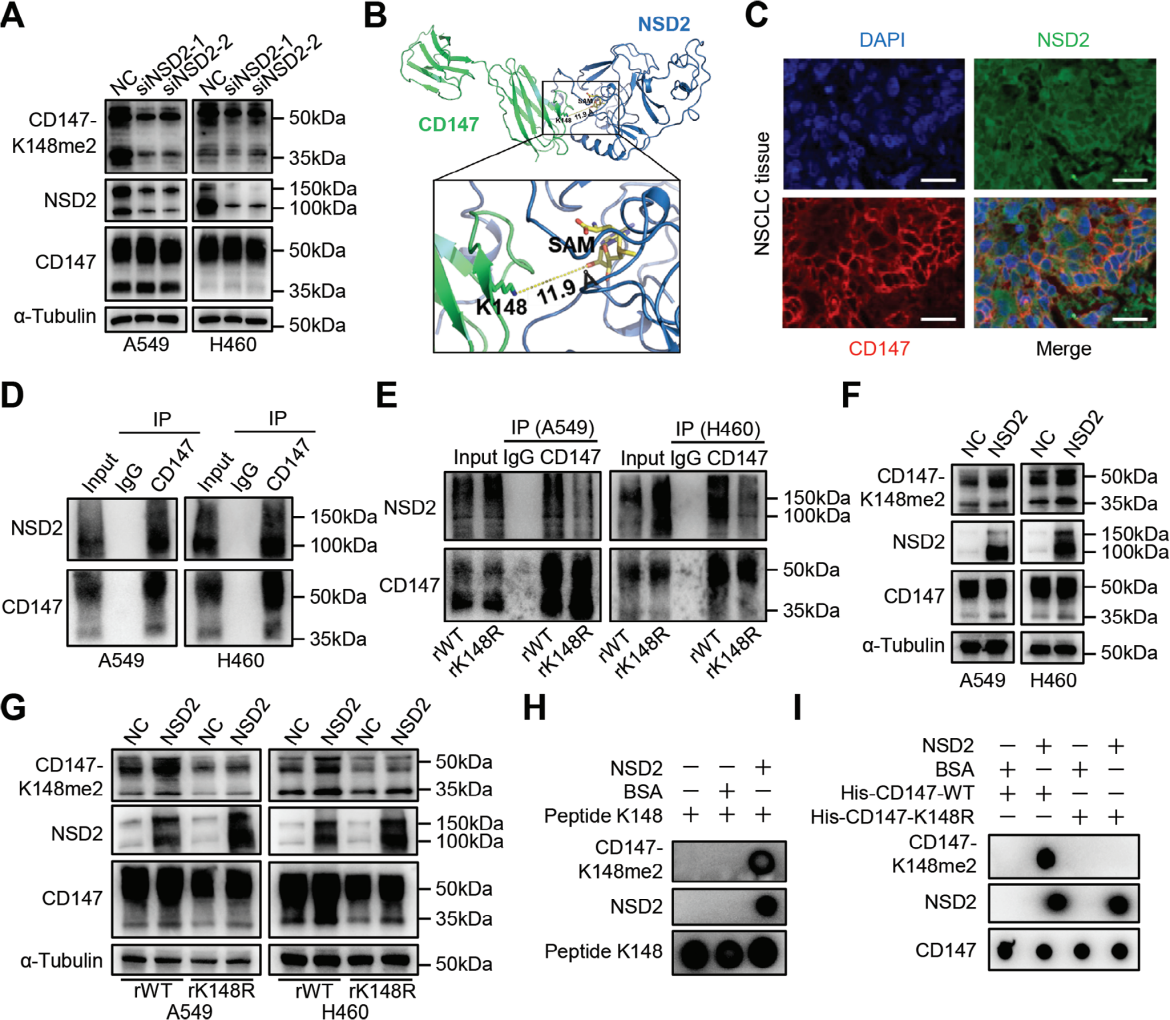
NSD2 acts on CD147 to generate CD147‐K148me2. A) The levels of CD147‐K148me2 and CD147 were determined by western blot in NSD2 knockdown A549 and H460 cells. B) Structural model of the CD147‐NSD2 complex in a cartoon diagram, with CD147 in green and the NSD2 SET domain in blue. CD147‐K148 and NSD2‐SAM are shown as sticks, and the distance between them is indicated by yellow dashed line. C) The co‐localization of CD147 (red) and NSD2 (green) in NSCLC tissue was detected by immunofluorescence staining, scale bar, 25 µm. D) The interaction between CD147 and NSD2 was confirmed in A549 and H460 cells by co‐IP assays, and IgG was used as a negative control. E) The interaction between CD147 and NSD2 was analyzed in the rWT and rK148R cells (A549 and H460) by co‐IP assays, and IgG was used as a negative control. F) The expression of CD147, NSD2, and CD147‐K148me2 was determined in the NSD2‐overexpressing cells (A549 and H460). G) The expression of CD147, NSD2, and CD147‐K148me2 was determined in the NSD2‐overexpressing rWT and rK148R cells (A549 and H460). H) An in vitro methyltransferase assay was performed with the K148 peptide and recombinant NSD2 protein co‐incubated in methyltransferase buffer containing the methyl donor SAM. The di‐methylation of the K148 peptide was detected by an anti‐CD147‐K148me2 antibody (12C8). I) An in vitro methyltransferase assay was performed with recombinant NSD2 protein and His‐CD147‐WT or His‐CD147‐K148R co‐incubated in methyltransferase buffer containing the methyl donor SAM. The di‐methylation of the CD147 protein was detected by an anti‐CD147‐K148me2 antibody (12C8).

Given the importance of CD147‐K148me2 in NSCLC progression, we investigated the specific methyltransferase for CD147‐K148 to further elucidate this modification process. siRNAs targeting the corresponding methyltransferases (SETD1A, SETDB1, SETD6, SETD7, G9A, EZH2, SUV39H2, NSD1, and NSD2) were synthesized and transfected into H460 cells. Then, the expression of CD147 and CD147‐K148me2 was determined by western blot. Interestingly, the transfection of siRNAs did not affect CD147 expression but specifically reduced the level of CD147‐K148me2 in the NSD2 knockdown groups (**Figure** **2**A; Figure S4A,B, Supporting Information). A similar result was also obtained in A549 cells (Figure 2A), suggesting that NSD2 is a potential candidate methyltransferase for CD147‐K148. The docking model showed that the di‐methylation site Lys148 of CD147 was positioned toward the catalytic pocket of the methyltransferase NSD2, where the methyl donor SAM was located (Figure 2B). The distance between the side chain of Lys148 and SAM was 11.9 Å, indicating close proximity for the methylation reaction. The free energy of binding (ΔG) of the complex was calculated to be −8 kcal mol⁻^1^ by PRODIGY, suggesting a stable and favorable interaction. These results suggest a possible catalytic mechanism for the di‐methylation of CD147 by NSD2. Moreover, co‐localization of CD147 and NSD2 was observed in NSCLC tissues (Figure 2C), and a co‐immunoprecipitation (co‐IP) assay demonstrated an interaction between CD147 and NSD2 in A549 and H460 cells (Figure 2D). Then, stable CD147 knockdown cell lines (A549/H460‐shCD147) were generated by lentiviral infection (Figure S5A,B, Supporting Information), and lentiviruses containing wild‐type CD147 (CD147‐WT) and mutant CD147 (CD147‐K148R) were transfected into A549/H460‐shCD147 cells to generate CD147‐reexpressing cell lines (A549/H460‐rWT and rK148R, respectively) (Figure S5C,D, Supporting Information). Subsequently, the CD147‐NSD2 interaction was investigated in the A549/H460‐rWT and rK148R cell lines. Compared to that in the rWT cells, a weakened interaction between CD147 and NSD2 was found in the rK148R cells (Figure 2E). NSD2 overexpression increased the CD147‐K148me2 levels in A549 and H460 cells (Figure 2F). NSD2 was also introduced to the rWT and rK148R cells, and the results demonstrated that NSD2 increased CD147‐K148me2 levels in the rWT cells, while no change was observed in the rK148R cells (Figure 2G). In vitro methyltransferase assays revealed that the peptide K148 and His‐CD147‐WT protein were methylated by NSD2 in methyltransferase buffer, while this modification was not found in the His‐CD147‐K148R group (Figure 2H,I). These findings indicate that the methyltransferase NSD2 catalyzes the Lys148 residue of CD147 to generate CD147‐K148me2.

### 2.3 CD147‐K148me2 Promotes CCL5 Secretion and M2‐Like TAM Migration in NSCLC

**Figure 3:**
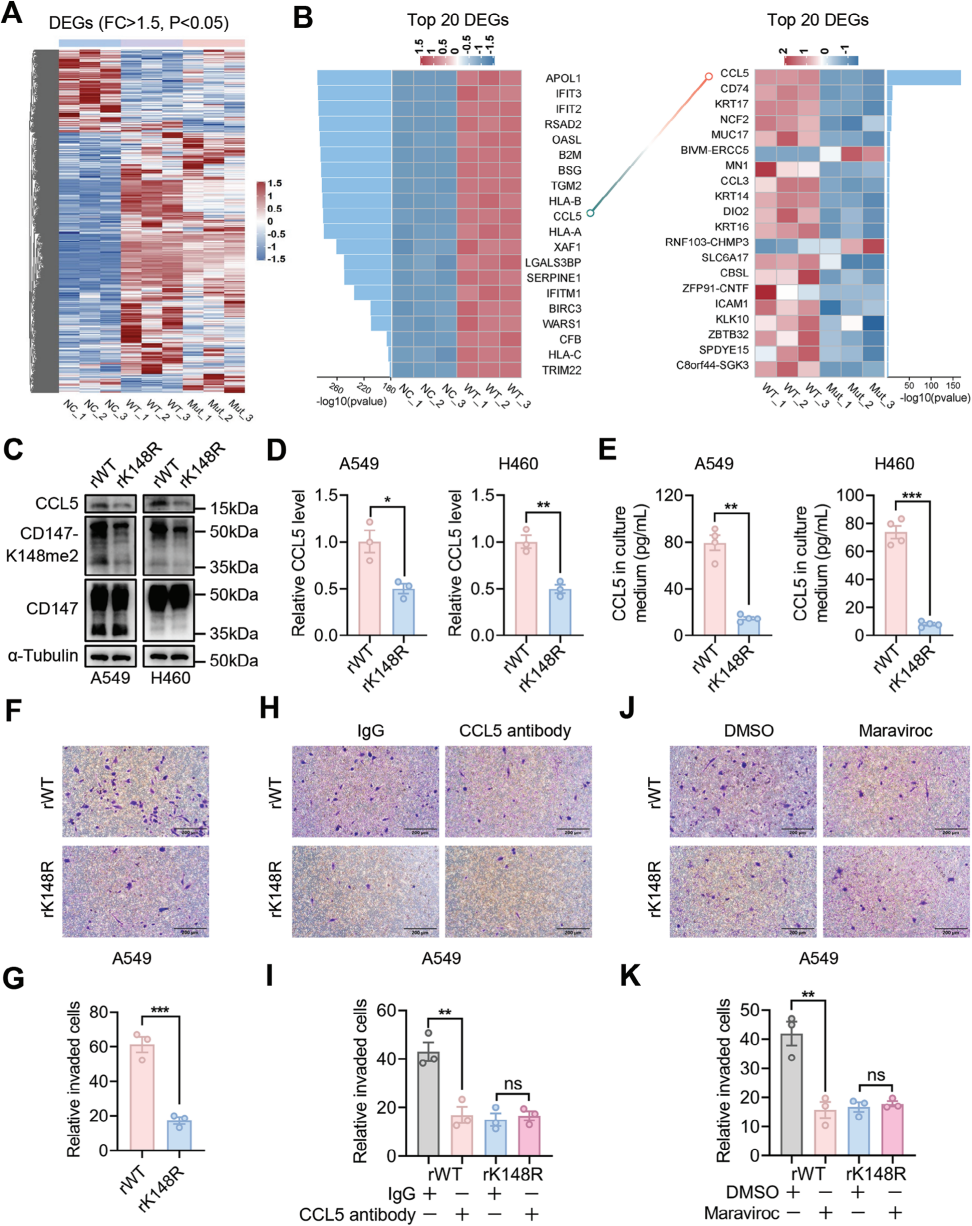
CD147‐K148me2 increases CCL5 secretion and M2‐like TAM migration. A) DEGs (fold change (FC) >1.5 and P < 0.05) among the control (NC), CD147‐WT (WT)‐ and CD147‐K148R (Mut)‐overexpressing A549 cells were identified by RNA‐seq. B) The top 20 DEGs in the NC versus WT and WT versus Mut comparisons were displayed, and CCL5 was identified as the gene with the most significant difference between the WT and Mut groups. C) The expression levels of CD147, CD147‐K148me2, and CCL5 were determined in the rWT and rK148R cells (A549 and H460) by western blot analyses. D) The mRNA level of CCL5 was determined in the rWT and rK148R cells (A549 and H460) by RT‐PCR (*P < 0.05; **P < 0.01; n = 3). E) CCL5 levels in the culture media of the rWT and rK148R cells (A549 and H460) were determined by ELISAs (**P < 0.01; ***P < 0.001; n = 4). F,G) A cell chemotaxis assay was conducted by co‐incubation of M2‐like macrophages and A549‐rWT or A549‐rK148R cells, scale bar, 200 µm (F), and the quantitative analysis was conducted via three independent experiments (***P < 0.001; n = 3) (G). H,I) A cell chemotaxis assay was conducted by co‐incubation of M2‐like macrophages and A549‐rWT or A549‐rK148R cells with IgG or CCL5 antibody, scale bar, 200 µm (H), and the quantitative analysis was conducted via three independent experiments (**P < 0.01; ns, not significant; n = 3) (I). J,K) A cell chemotaxis assay was conducted by co‐incubation of M2‐like macrophages and A549‐rWT or A549‐rK148R cells with DMSO or Maraviroc, scale bar, 200 µm (J), and the quantitative analysis was conducted via three independent experiments (**P < 0.01; ns, not significant; n = 3) (K).

To investigate the role of CD147‐K148me2 in NSCLC progression, we performed proliferation and migration assays, and the results revealed no obvious differences between the rWT and rK148R groups (Figure S6A–D, Supporting Information). Then, we conducted RNA‐seq analysis using CD147‐WT (WT)‐ and CD147‐K148R (Mut)‐overexpressing A549 cells, with A549 cells transfected with a mock plasmid (NC) serving as the control (**Figure** **3**A). Genes with a fold change >1.5 and P<0.05 were defined as differentially expressed genes (DEGs). Among the DEGs, CCL5 was the most significant DEG between the WT and Mut groups and was also among the top 10 DEGs between the NC and WT groups (Figure 3B). These results indicated that CD147‐K148me2 promoted CCL5 secretion in NSCLC cells. Western blot analysis revealed a decreasing trend in CD147‐K148me2 and CCL5 expression in the rK148R group compared with the rWT group (Figure 3C), and this result was also verified in A549 and H460 cells by RT‐PCR (Figure 3D). Since CCL5 is a secreted protein, the content of CCL5 in the culture medium was determined by ELISAs, and similar results were obtained (Figure 3E). Taken together, these findings indicate that this modification promotes CCL5 generation in NSCLC cells.

Subsequently, Gene Ontology (GO), Kyoto Encyclopedia of Genes and Genomes (KEGG) analysis, and gene set enrichment analysis (GSEA) were performed using the above‐mentioned RNA‐seq data. The top 20 GO terms of DEGs in the WT versus Mut and NC versus WT groups included “cell chemotaxis”, “macrophage chemotaxis”, “immune response”, “response to cytokine”, and “cytokine‐mediated signaling pathway” (Figure S7A,B, Supporting Information). GSEA also identified “chemokine activity”, “CCR chemokine receptor binding”, “monocyte chemotaxis”, and “response to chemokine” (Figure S7C,D, Supporting Information). Similar results were obtained via KEGG analysis (Figure S8A–D, Supporting Information). These results indicate that CD147‐K148me2 is closely related to immune cell chemotaxis and the immune response.

CCL5 is an important chemotactic cytokine that promotes immune cell migration via the CCL5/CCR5 axis.^[^ ^22^ ^]^ Then, the NSCLC single‐cell RNA sequencing data were analyzed, and CCR5 was found to be present in monocytes and macrophages (Figure S9A,B, Supporting Information). Data from the TISIDB database showed a significant correlation between CCL5 expression and macrophage infiltration in LUAD and LUSC (Figure S9C, Supporting Information). Previous studies showed that the CCL5/CCR5 axis contributed to tumor progression by promoting the formation of an immunosuppressive TME, including M2‐TAM chemotaxis.^[^ ^22^, ^23^ ^]^ Next, multi‐color immunofluorescence staining was performed using NSCLC tissues (Figure S9D, Supporting Information), and the results showed that the high CD147‐K148me2 group had more infiltrated CD68+CD206+ M2‐TAMs than did the low CD147‐K148me2 group (Figure S9E, Supporting Information). A significant correlation was observed between CD147‐K148me2 expression and M2‐TAM infiltration in NSCLC tissues (Figure S9F, Supporting Information).

Subsequently, the chemotaxis of M2‐TAMs mediated by CD147‐K148me2‐induced CCL5 secretion was evaluated by a Transwell assay. The THP‐1 cells were differentiated into M2‐like macrophages with increased expression of CD163 and CD206 (Figure S10A,B, Supporting Information). Chemotaxis assays revealed that M2‐like macrophages exhibited greater migration in the rWT group than in the rK148R group (Figure 3F,G; Figure S10C,D, Supporting Information). The addition of CCL5 antibody to the culture medium inhibited M2‐like macrophage migration in the rWT group, whereas no significant alteration was observed in the rK148R group (Figure 3H,I; Figure S10E,F, Supporting Information). Similarly, CCR5 inhibition in Maraviroc‐treated M2‐like macrophages yielded comparable results in both the rWT and rK148R groups (Figure 3J,K; Figure S10G,H, Supporting Information). These findings indicate that CD147‐K148me2 facilitates CCL5 secretion and increases M2‐like TAM migration via CCL5/CCR5 axis‐mediated cellular crosstalk, suggesting that CD147‐K148me2 contributes to the immunosuppressive TME in NSCLC.

### 2.4 CD147‐K148me2 Facilitates CCL5 Secretion in NSCLC Cells by Activating CyPA‐CD147‐p38 Signaling

**Figure 4:**
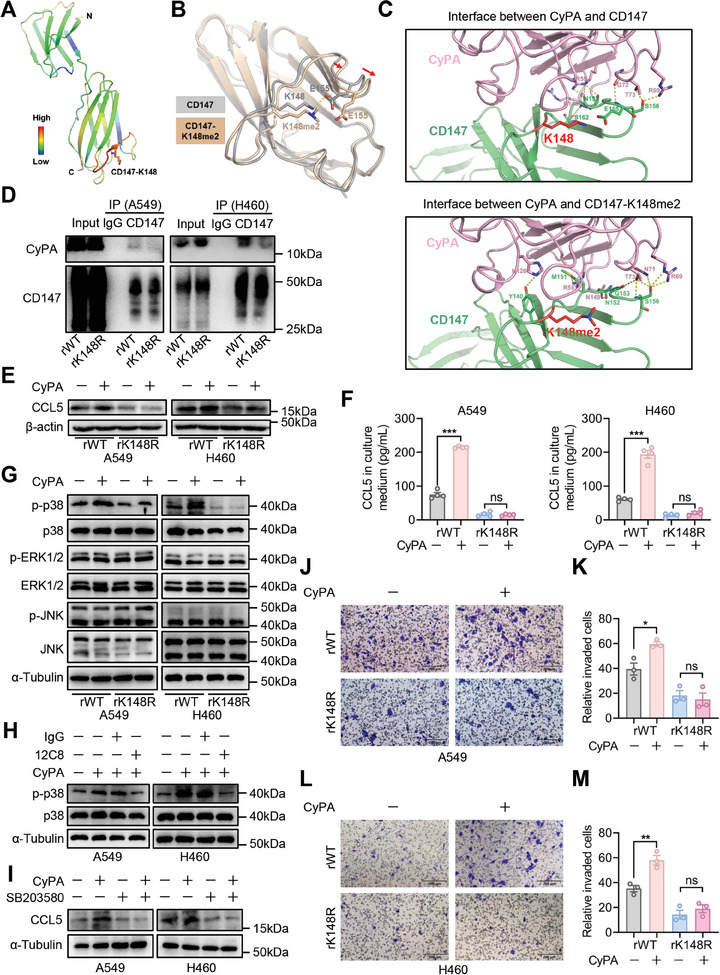
CD147‐K148me2 facilitates CCL5 secretion in NSCLC cells by activating CyPA‐CD147‐p38 signaling. A) Temperature factor (B‐factor) distribution and K148 location of the CD147 molecule (PDB ID: 3B5H). B‐factor values range from blue (low B‐factor) to red (high B‐factor). B) Structural superposition of CD147 (gray) and CD147‐K148me2 (orange) based on their respective complex docking models. Arrows indicate local conformational changes induced by di‐methylation, and yellow dashed lines indicate polar contacts formed between amino acid residues. C) Close‐up view of the interaction interfaces of the docked complex model between CyPA (pink) and CD147 (green) without (upper) or with (lower) K148me2. K148 (red) and amino acid residues involved in hydrogen bond formation are shown as sticks. Hydrogen bonds are represented by yellow dashed lines. D) The interaction between CD147 and CyPA was analyzed in the rWT and rK148R cells (A549 and H460) by co‐IP assays, and IgG was used as a negative control. E) CCL5 expression was determined in rWT and rK148R cells (A549 and H460) with or without recombinant CyPA stimulation by western blot. F) CCL5 levels in the culture media of the rWT and rK148R cells (A549 and H460) treated with or without recombinant CyPA were analyzed by ELISAs (***P < 0.001; ns, not significant; n = 4). G) The levels of ERK1/2, p‐ERK1/2, p38, p‐p38, JNK, and p‐JNK were determined in the rWT and rK148R cells (A549 and H460) with or without recombinant CyPA stimulation by western blot. H) The levels of p38 and p‐p38 were determined in the A549 and H460 cells with or without recombinant CyPA stimulation or 12C8 blockade, and IgG was used as a negative control. I) CCL5 levels were analyzed in the A549 and H460 cells treated with or without recombinant CyPA or SB203580 by western blot. J,K) A cell chemotaxis assay was conducted by co‐incubation of M2‐like macrophages and A549‐rWT or A549‐rK148R cells with or without recombinant CyPA stimulation, scale bar, 200 µm (J), and the quantitative analysis was conducted using three independent experiments (*P < 0.05; ns, not significant; n = 3) (K). L,M) A cell chemotaxis assay was conducted by co‐incubation of M2‐like macrophages and H460‐rWT or H460‐rK148R cells with or without recombinant CyPA stimulation, scale bar, 200 µm (L), and the quantitative analysis was conducted using three independent experiments (**P < 0.01; ns, not significant; n = 3) (M).

The above findings revealed a positive correlation between CD147‐K148me2 levels and CCL5 secretion in NSCLC cells. However, the underlying mechanism responsible for the CD147‐K148me2‐mediated increase in CCL5 was still unknown. Previous studies have reported that CD147, an important inflammation‐related molecule, facilitates the secretion of cytokines and chemokines via CyPA/CD147‐activated intracellular signal delivery, including ERK1/2, p38, and JNK mitogen‐activated protein kinase (MAPK) signaling pathway.^[^ ^24^, ^25^ ^]^ Thus, we propose that CD147‐K148me2 promotes CCL5 secretion via CyPA/CD147‐activated MAPK signaling. Accordingly, we investigated the effect of CD147‐K148me2 on the interaction between CyPA and CD147. The Lys148 residue is located in the loop region that connects two β strands of CD147, and the B‐factor distribution based on the CD147 crystal structure showed that this loop had the highest B‐factor value, indicating its high structural flexibility (**Figure** **4**A). To further explore the structural mechanism by which CD147‐K148me2 leads to altered binding with CyPA, we constructed structural models of CyPA in complex with Lys148‐di‐methylated and unmethylated CD147 (Figure S11A, Supporting Information) using a strategy based on molecular docking and short‐time dynamic optimization. As shown in Figure 4B, the side chain of unmodified CD147‐K148 formed a polar interaction with CD147‐E155 on the opposite side of the loop, which stabilized the loop region to some extent. This di‐methylation caused an increase in the volume of the Lys148 side chain, disrupted the polar interaction with CD147‐E155, and thereby increased the overall flexibility of the loop, which facilitated more extensive interactions with CyPA. Further analysis revealed that compared with the unmodified CD147‐CyPA complex, the di‐methylated CD147‐CyPA complex formed more hydrogen bonds at the interface and had a larger interaction area and a lower free energy (Figure 4C; Figure S11B,C, Supporting Information). These findings indicated that this di‐methylation increased the binding capacity of CyPA for CD147. Co‐IP assays were also conducted to identify the interaction of the two proteins. Compared to that in the rK148R group, the CyPA‐CD147 interaction increased in the rWT group (Figure 4D), which verified the structural mechanism by which CD147‐K148me2 increased the affinity of CyPA for CD147.

Subsequently, recombinant CyPA protein was added to the culture medium of the rWT and rK148R cells, and the expression level of CCL5 in the rWT group significantly increased under CyPA stimulation, while no change was observed in the rK148R group (Figure 4E). Similar results were also obtained in ELISAs of secreted CCL5 in culture medium (Figure 4F). Moreover, the activation of MAPKs was identified by western blot, and the results showed that CyPA stimulation increased the phosphorylation of p38, while little change in the phosphorylation of ERK1/2 and JNK was found in the rWT group. However, the phosphorylation of ERK1/2, p38, and JNK was not obviously altered in the rK148R group under CyPA stimulation (Figure 4G; Figure S10I, Supporting Information). In A549 and H460 cells, CyPA promoted the phosphorylation of p38, which was inhibited by 12C8 blockade (Figure 4H). Similarly, the CyPA‐mediated increase in CCL5 levels was inhibited by a p38 inhibitor (SB203580) (Figure 4I). These findings indicate that CD147‐K148me2 promotes the CyPA‐CD147 interaction and increases CCL5 secretion by activating p38 signaling. A chemotaxis assay was also conducted in rWT and rK148R cells after CyPA addition, and the results showed that CyPA stimulation increased the migration of M2‐like macrophages in the rWT group, while no significant change was found in the rK148R group (Figure 4J–M). Taken together, these findings indicate that CD147‐K148me2 facilitates CCL5 secretion in NSCLC cells and increases M2‐like macrophage migration by activating CyPA‐CD147‐p38 signaling.

### 2.5 CD147‐K148me2‐Mediated CCL5 Secretion Is Involved in the Regulation of ZBTB32

**Figure 5:**
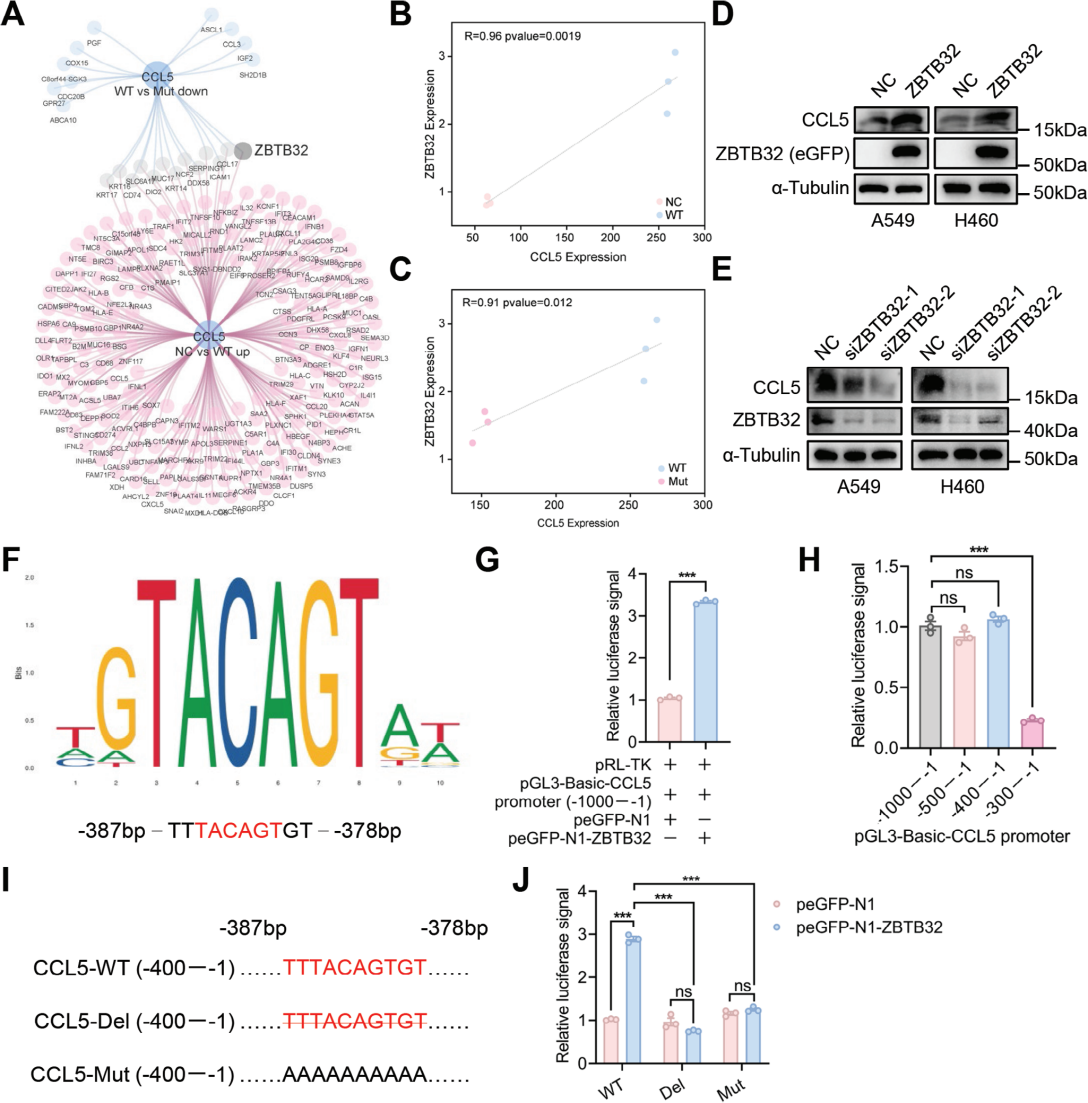
CCL5 expression is closely related to the regulation of the transcription factor ZBTB32. A) The transcription factors associated with CCL5 expression were screened in the WT versus Mut and NC versus WT groups using RNA‐seq analysis. Thirteen DEGs were shared between the two groups, and ZBTB32 was the only transcription factor. B) The correlation between ZBTB32 and CCL5 expression was analyzed in the NC and WT groups using RNA‐seq data (R = 0.96, P = 0.0019). C) The correlation between ZBTB32 and CCL5 expression was analyzed in the WT and Mut groups using RNA‐seq data (R = 0.91, P = 0.012). D) CCL5 expression was determined in the ZBTB32‐overexpressing cells (A549 and H460) by western blot. E) CCL5 expression was determined in the ZBTB32 knockdown cells (A549 and H460) by western blot. F) The binding site of ZBTB32 to the CCL5 gene promoter was analyzed with a relative profile score threshold >90% using JASPAR. G) Dual‐luciferase reporter assays were performed after co‐transfecting of the pGL3‐Basic‐CCL5 promoter (−1000–−1), pRL‐TK, and plasmid ZBTB32 or its control into HEK293T cells. The relative luciferase signal was detected using a Dual‐Luciferase® Reporter Assay System (***P < 0.001; n = 3). H) Dual‐luciferase reporter assays were performed after co‐transfecting of pGL3‐Basic with different truncated sequences of the CCL5 gene promoter, pRL‐TK, and the ZBTB32 plasmid into HEK293T cells. The relative luciferase signal was detected using a Dual‐Luciferase® Reporter Assay System (***P < 0.001; ns, not significant; n = 3). I) The predicted binding sequence of the CCL5 gene promoter (−387bp–TTTACAGTGT–−378 bp) was deleted and mutated to generate the new plasmids CCL5‐Del (−400–−1) and CCL5‐Mut (−400–−1), respectively. J) Dual‐luciferase reporter assays were performed after co‐transfecting of CCT‐WT (CCL5‐Del/CCL5‐Mut) with pRL‐TK and the plasmid ZBTB32 or its control into HEK293T cells. The relative luciferase signal was detected using a Dual‐Luciferase® Reporter Assay System (***P < 0.001; ns, not significant; n = 3).

p38 kinase signaling was shown to regulate the biological functions of different cell types by trans‐locating to the nucleus and affecting the expression of transcription factors and their corresponding downstream genes.^[^ ^26^ ^]^ Therefore, RNA‐seq data were used to screen for transcription factors associated with CCL5 expression. DEGs with a positive correlation with CCL5 (R > 0.9 and P < 0.05) were selected from the WT versus Mut and NC versus WT comparisons. Thirteen DEGs were shared between the two groups, and ZBTB32 was the only transcription factor (**Figure** **5**A–C). The positive correlation between CCL5 and ZBTB32 in NSCLC was also verified using other databases, including the TISIDB database (R = 0.615, P < 2.2e‐16, LUAD; R = 0.518, P < 2.2e‐16, LUSC), GEPIA database (R = 0.59, P = 6.5e‐47, LUAD; R = 0.4, P = 1.2e‐19, LUSC), and TIMER database (R = 0.629, P = 3.65e‐58, LUAD; R = 0.526, P = 4.89e‐37, LUSC) (Figure S12A–C, Supporting Information). Moreover, data from the TISIDB database showed that macrophage infiltration was closely associated with ZBTB32 expression in LUAD (R = 0.34, P = 2.67e‐15) and LUSC (R = 0.427, P < 2.2e‐16) (Figure S12D, Supporting Information). Further study revealed that ZBTB32 overexpression increased CCL5 levels in A549 and H460 cells (Figure 5D). Conversely, silencing ZBTB32 had the opposite effect, leading to a decrease in CCL5 levels (Figure 5E). These results suggested that ZBTB32 likely regulated the transcription of the CCL5 gene.

Then, the binding sites of ZBTB32 to the CCL5 gene promoter were analyzed with a relative profile score threshold >90% using JASPAR. The sequence of the CCL5 gene promoter (−387bp–TTTACAGTGT–−378 bp) was considered a candidate binding site. A firefly luciferase reporter vector containing the CCL5 promoter (−1000–−1) was created and transfected into HEK293T cells with pRL‐TK with renilla luciferase and the plasmid ZBTB32 or its control. The dual‐luciferase reporter assay showed that the CCL5 gene promoter exhibited a greater luciferase signal in the ZBTB32 group than in the control group (Figure 5F,G). To identify the specific binding site of ZBTB32 with the CCL5 gene promoter, we generated truncated sequences of the CCL5 gene promoter, and the results showed that the luciferase signal decreased significantly in the truncated plasmid (−300–−1) group, suggesting that the potential binding site was probably located at positions −400–−300 of the CCL5 gene promoter (Figure 5H). The predicted binding sequence of the CCL5 gene promoter (−387bp–TTTACAGTGT–−378 bp) was deleted and mutated to generate the new plasmids CCL5‐Del (−400–−1) and CCL5‐Mut (−400–−1), respectively (Figure 5I). Compared to those in the CCL5‐WT group, the luciferase signals decreased significantly in both the CCL5‐Del and CCL5‐Mut groups (Figure 5J), indicating that ZBTB32 interacted with this region. These findings indicate that the −387–−378 region of the CCL5 gene promoter is important for the binding of ZBTB32.

**Figure 6:**
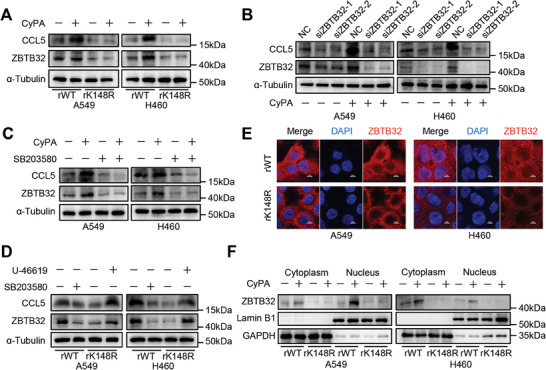
CD147‐K148me2‐mediated CCL5 secretion is involved in the regulation of ZBTB32. A) The expression levels of ZBTB32 and CCL5 were analyzed in the rWT and rK148R cells (A549 and H460) with or without recombinant CyPA stimulation by western blot. B) The expression of CCL5 was analyzed in the ZBTB32 knockdown cells (A549 and H460) with or without recombinant CyPA stimulation by western blot. C) The expression of ZBTB32 and CCL5 in the A549 and H460 cells treated with or without recombinant CyPA or SB203580 was analyzed by western blot. D) The expression levels of ZBTB32 and CCL5 were analyzed in the rWT and rK148R cells (A549 and H460) treated with SB203580 or U‐46619 by western blot. E) The localization of the ZBTB32 protein (red) was identified in the rWT and rK148R cells (A549 and H460) using immunofluorescence staining, scale bar, 5 µm. F) Nuclear and cytoplasmic protein extraction assays were performed to determine the nuclear expression and cytoplasmic expression of ZBTB32 with or without recombinant CyPA stimulation in the rWT and rK148R cells (A549 and H460). Lamin B1 and GAPDH were selected as controls for nuclear protein and cytoplasmic protein, respectively.

To validate the regulatory effect of CD147‐K148me2 on ZBTB32, we examined the expression of ZBTB32 and CCL5 in rWT and rK148R cells with or without CyPA stimulation. The results showed that CyPA supplementation increased the expression of both ZBTB32 and CCL5 in the rWT group, while no obvious change was observed in the rK148R group (**Figure** **6**A). ZBTB32 knockdown in A549 and H460 cells inhibited the expression of CCL5, and this change was not reversed by CyPA supplementation (Figure 6B). In addition, the administration of SB203580 inhibited the CyPA‐mediated upregulation of ZBTB32 and CCL5 in A549 and H460 cells (Figure 6C). In the rWT group, SB203580 inhibited ZBTB32 and CCL5 expression by affecting p38 signaling. Supplementation with a p38 activator (U‐46619) reversed the expression of ZBTB32 and CCL5 in the rK148R cells (Figure 6D). Immunofluorescence staining showed that ZBTB32 expression in the cell nucleus was greater in the rWT group than in the rK148R group (Figure 6E), and these results were also verified by nuclear and cytoplasmic protein extraction assays (Figure 6F). Meanwhile, CyPA stimulation promoted ZBTB32 expression and facilitated its translocation to the nucleus (Figure 6F). Taken together, these findings indicate that the CyPA‐CD147‐K148me2 interaction increases CCL5 secretion by activating the p38‐ZBTB32 axis in NSCLC cells.

### 2.6 CD147‐K148me2 Promotes NSCLC Progression by Inducing M2‐Like TAM Infiltration, Which Is Inhibited by an Anti‐CD147‐K148me2 Antibody

**Figure 7:**
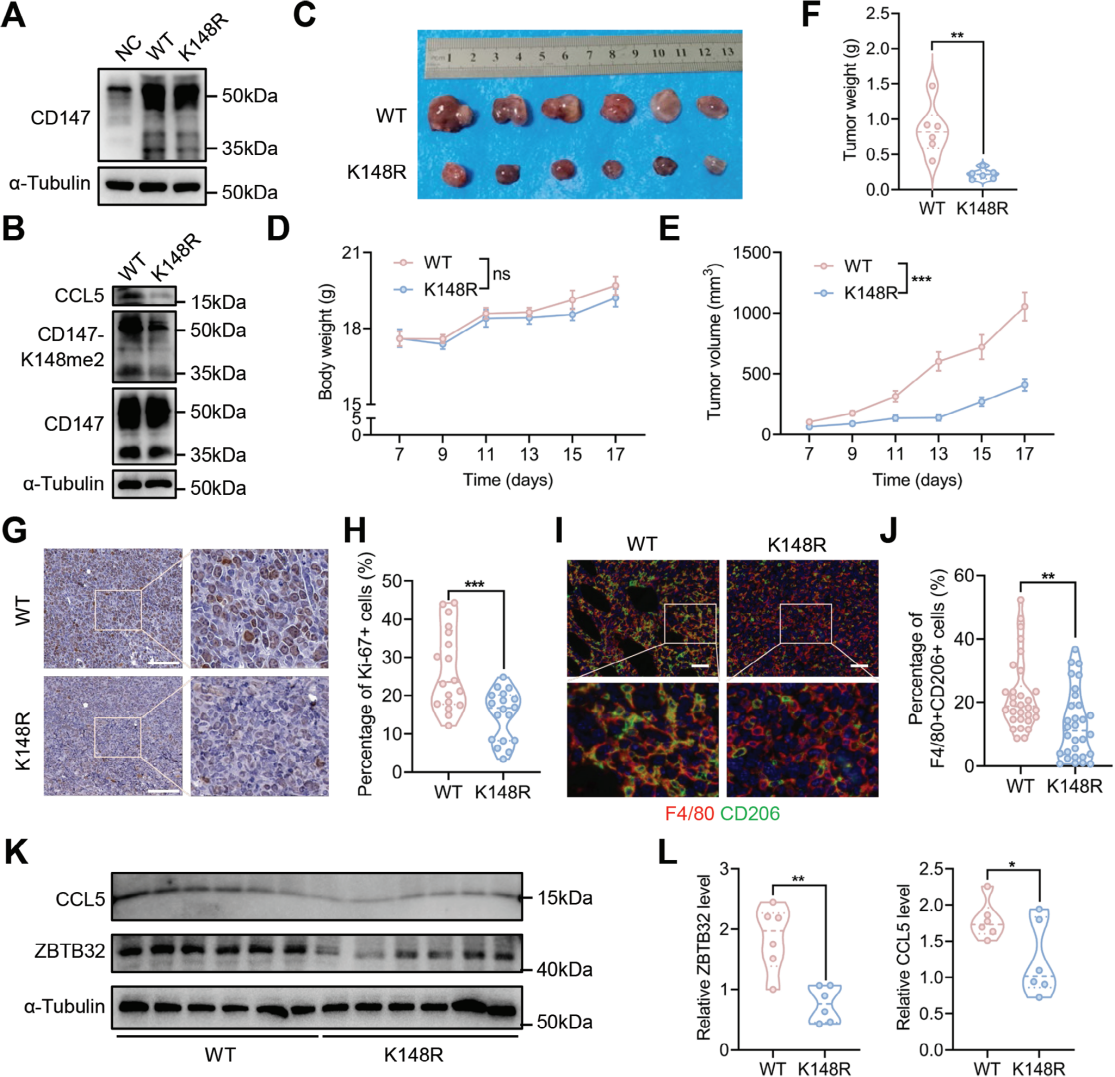
CD147‐K148me2 promotes NSCLC progression by inducing M2‐like TAM infiltration. A) The expression of CD147 was determined in the LLC‐WT and LLC‐K148R cells by western blot. B) The expression levels of CD147, CD147‐K148me2, and CCL5 were determined in the LLC‐WT and LLC‐K148R cells by western blot. C) Image of tumor masses from the LLC‐WT (n = 6) and LLC‐K148R (n = 6) groups. D) The body weights of the mice in the LLC‐WT and LLC‐K148R groups were measured every two days (ns, not significant; n = 6). E,F) The volume (E) and weight (F) of the tumor masses were measured (**P < 0.01; ***P < 0.001; n = 6). G,H) The percentage of Ki‐67+ cells was determined in tumor tissues from the LLC‐WT and LLC‐K148R groups by IHC staining, scale bar, 100 µm (G). Three fields from each mouse were used for statistical analysis (n = 18 for each group; ***P < 0.001) (H). I,J) F4/80+CD206+ cell infiltration was determined in tumor tissues from the LLC‐WT and LLC‐K148R groups by multi‐color immunofluorescence staining, scale bar, 50 µm (I). Five fields from each mouse were used for statistical analysis (n = 30 for each group; **P < 0.01) (J). K,L) The expression of ZBTB32 and CCL5 in tumor tissues was determined by western blot (K), and statistical analysis was conducted (n = 6 for each group; *P < 0.05; **P < 0.01) (L).

To further investigate the role of CD147‐K148me2 in NSCLC progression, we performed in vivo analyses with a cell line‐derived xenograft (CDX) model. Given the high similarity between human NSD2 and mouse NSD2, lentiviruses encoding human CD147 (WT or K148R) were introduced into mouse LLC cells to generate LLC‐WT/K148R cells (**Figure** **7**A). Western blot analysis showed that the levels of CD147‐K148me2 and CCL5 were greater in the WT group than in the K148R group (Figure 7B), indicating that mouse NSD2 catalyzes the generation of CD147‐K148me2 from human CD147 and that this modification increased the level of CCL5 in LLC cells. Therefore, the LLC‐WT/K148R cell lines were suitable for in vivo testing. For the CDX model, the mice were injected subcutaneously with LLC‐WT (n = 6) or LLC‐K148R (n = 6) cells. At the experimental endpoint, the tumor masses were collected (Figure 7C), and no significant difference in body weight was observed between the WT and K148R groups (Figure 7D). However, the tumor volume in the WT group was greater than that in the K148R group (Figure 7E), and a similar trend was observed for tumor weight in the two groups (Figure 7F). IHC staining revealed a greater percentage of Ki‐67+ cells in the WT group than in the K148R group (Figure 7G,H). Multi‐color immunofluorescence staining revealed high infiltration of F4/80+CD206+ cells in the WT group, indicating that CD147‐K148me2 promoted M2‐like TAM infiltration (Figure 7I,J). The expression of ZBTB32 and CCL5 was also determined by western blot analysis using tumor masses, and the mutation of di‐methylated Lys148 inhibited ZBTB32 and CCL5 expression (Figure 7K,L), which was consistent with the results in A549 and H460 cells. These findings demonstrate that CD147‐K148me2 promotes NSCLC progression by increasing M2‐like TAM infiltration.

**Figure 8:**
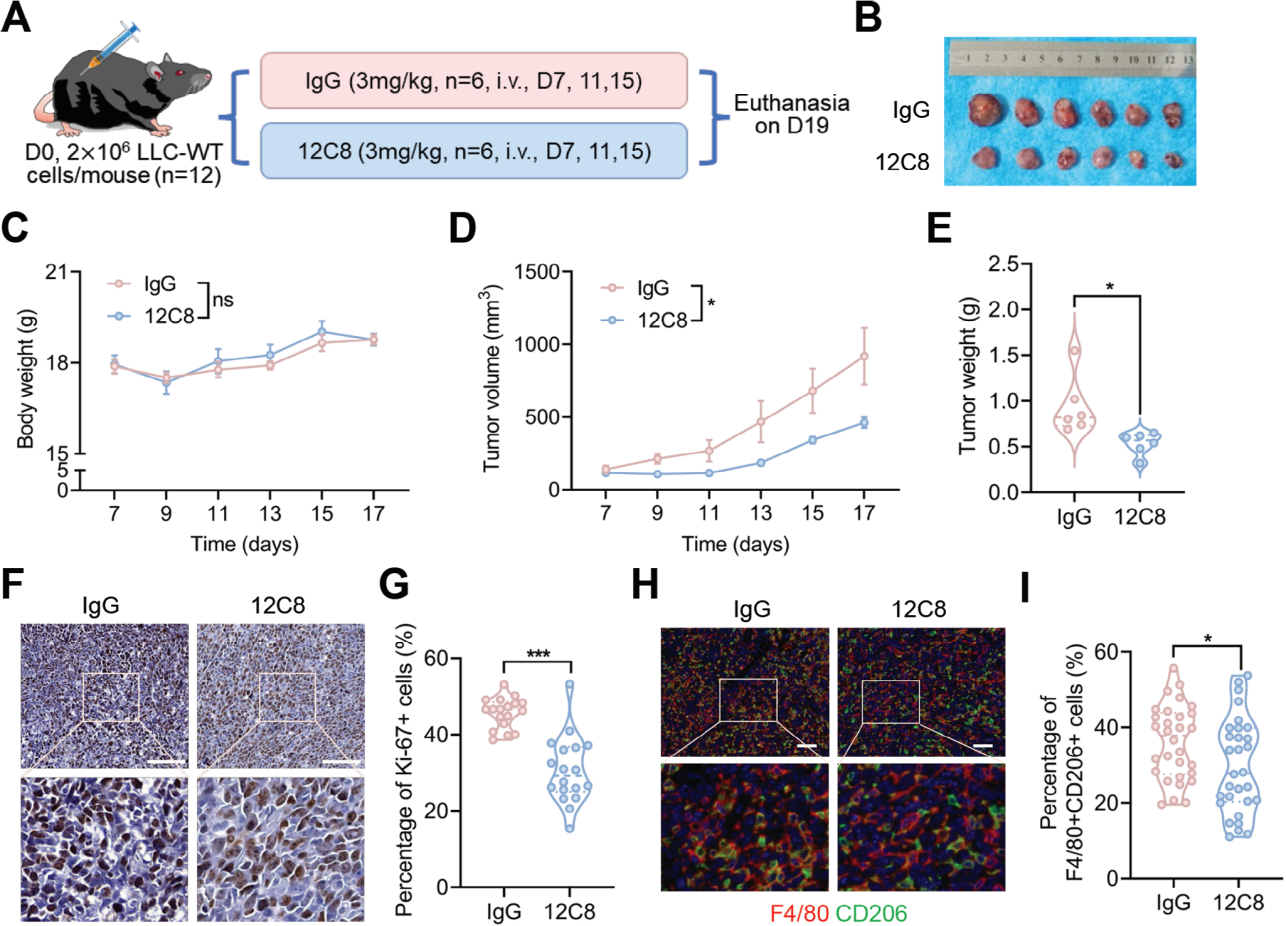
The anti‐CD147‐K148me2 antibody inhibits NSCLC immunosuppression and progression by interrupting the CyPA‐CD147 interaction. A) The flow chart for tumor treatment with 12C8 antibody. B) Image of tumor masses from the IgG (n = 6) and 12C8 (n = 6) groups. C) The body weights of the mice in the IgG and 12C8 groups were measured every two days (ns, not significant; n = 6). D,E) The volume (D) and weight (E) of the tumor masses were measured (*P < 0.05; n = 6). F,G) The percentage of Ki‐67+ cells was determined in tumor tissues from the IgG and 12C8 groups by IHC staining, scale bar, 100 µm (F). Three fields from each mouse were used for statistical analysis (n = 18 for each group; ***P < 0.001) (G). H,I) The infiltration of F4/80+CD206+ cells was determined in tumor tissues from the IgG and 12C8 groups by multi‐color immunofluorescence staining, scale bar, 50 µm (H). Five fields from each mouse were used for statistical analysis (n = 30 for each group; *P < 0.05) (I).

To investigate the efficacy of 12C8 in tumor treatment, we performed an in vivo test by intravenous administration of 12C8 (3 mg kg^−1^) three times, and an equal amount of IgG was used as a control (**Figure** **8**A). Following therapy, the tumor masses were collected (Figure 8B), and the body weights were not significantly different between the IgG and 12C8 groups (Figure 8C). Compared to the control, the administration of 12C8 significantly reduced the volume and weight of the tumor masses (Figure 8D,E). IHC staining demonstrated that the percentage of Ki‐67+ cells in the IgG group was greater than that in the 12C8 group (Figure 8F,G), suggesting that 12C8 treatment significantly inhibited tumor growth. Multi‐color immunofluorescence staining was performed using slides from both the IgG and 12C8 groups. In contrast to that in the IgG group, the percentage of F4/80+CD206+ cells was lower in the 12C8 group (Figure 8H,I), suggesting that 12C8 treatment significantly suppressed M2‐like TAM infiltration in tumor tissues. These findings indicate that targeting CD147‐K148me2 is a potential strategy for tumor treatment and lays the foundation for PTM‐targeted tumor therapy.

## 3 Discussion

NSCLC is a common disease that threatens human health. Identifying novel factors associated with NSCLC progression will directly contribute to tumor prognosis and treatment. Advances in proteomics research and LC‐MS/MS technology have led to increased focus on PTMs, and these PTMs have gained prominence as potential neoantigens or pathogenic factors that accelerate tumor evolution. CD147, a tumor‐associated antigen, has been reported to be an important pro‐tumorigenic factor and to participate in multi‐temporal and multi‐stage tumor development.^[^ ^17^, ^19^, ^27^, ^28^, ^29^ ^]^ In this study, the di‐methylation of CD147 at Lys148, which is catalyzed by the methyltransferase NSD2, was identified as a new tumor biomarker for NSCLC. Compared to that in para‐carcinoma tissues, CD147‐K148me2 was highly expressed in NSCLC tissues. High CD147‐K148me2 levels were closely associated with poor overall survival in NSCLC patients, especially in patients with advanced‐stage NSCLC, possibly because of the high expression of NSD2 in this stage.

Protein methylation plays a crucial role in tumor development and is involved in diverse biological processes, especially regulating protein‐protein interactions that contribute to abnormal signal transduction and disease development.^[^ ^30^, ^31^ ^]^ CyPA and CD147 are regarded as essential ligand and receptor that activate multiple oncogenic signaling pathways, and agents that interfere with the interaction between CD147 and CyPA have the potential to inhibit tumor progression.^[^ ^25^ ^]^ In our study, the di‐methylation of CD147 at Lys148 had a potential influence on protein conformation, which increased the binding capacity of CyPA to CD147, and in turn activated the p38‐ZBTB32 signaling, leading to increasing NSCLC cell‐derived CCL5 secretion. These findings identify the role of CD147‐K148me2 in enhancing CyPA‐mediated intracellular signal transduction and illustrate the pathogenesis of the CyPA/CD147 complex in tumor progression from the perspective of PTMs.

It has been reported that the immunosuppressive TME determines the anti‐tumor immune response to immunotherapy to some extent,^[^ ^32^ ^]^ which is usually caused by the intercellular crosstalk between immune cells and immune cells or between immune cells and other cells in the TME. In NSCLC, tumor cells educate macrophages to express MARCO and acquire an immunosuppressive phenotype by secreting IL‐37, which diminishes CD8+ T cell activities and killing capacity.^[^ ^33^ ^]^ Cancer‐associated fibroblasts interact with tumor‐infiltrating immune cells, reshaping the immunosuppressive TME via the secretion of different chemokines, cytokines, and other effector molecules.^[^ ^34^ ^]^ In our study, we found that CD147‐K148me2‐mediated CCL5 increase in NSCLC cells facilitated intercellular crosstalk between tumor cells and M2‐TAMs via the CCL5/CCR5 axis, leading to the infiltration of M2‐TAMs in NSCLC and tumor progression. Hence, the disruption of abnormal intercellular crosstalk in the TME is beneficial to reverse the immunosuppressive TME and enhance anti‐tumor immune response of tumor patients.

Currently, the strategies of modulating the TME have gained instant attention and have made a great progress in overcoming immunosuppression and triggering robust immunotherapeutic responses.^[^ ^35^, ^36^, ^37^, ^38^, ^39^ ^]^ In our study, a specific antibody 12C8 targeting CD147‐K148me2 was generated by the immunization with di‐methylated peptide. Importantly, the administration of 12C8 inhibited CD147‐K148me2‐mediated CCL5 increase and the CCL5/CCR5 axis‐dependent intercellular crosstalk between tumor cells and macrophages, which decreased the infiltration of M2‐TAMs in tumor tissues and showed a notable anti‐tumor effect in in vivo tests. These findings demonstrate the contribution of CD147‐K148me2 to NSCLC immunosuppression and indicate that this modification is a potential target for NSCLC treatment from the perspective of eradicating tumor immunosuppression, which is expected to increase the effect of cancer immunotherapy for patients with NSCLC.

In summary, our study identifies an important PTM, CD147‐K148me2, in NSCLC and reveals the role of CD147‐K148me2‐mediated intercellular crosstalk in NSCLC immunosuppression and progression, suggesting that targeting CD147‐K148me2 is a potential interventional strategy for NSCLC therapy.

## 4 Experimental Section

### Cell Lines

The cells were obtained from the American Type Culture Collection (A549, LLC, and THP‐1) and the Cell Bank of the Chinese Academy of Sciences (H460 and HEK293T), which were tested and authenticated by Beijing Microread Genetics (Beijing, China). Stable CD147 knockdown cell lines (A549/H460‐shCD147) were established by transfecting CD147 lentiviral shRNA (the laboratory), and lentiviruses (GENECHEM, China) containing human wild‐type CD147 (CD147‐WT) and mutant CD147 (CD147‐K148R) were transfected into A549/H460‐shCD147 and LLC cells to generate CD147‐reexpressing cell lines (A549/H460‐rWT and rK148R) and CD147‐overexpressing cell lines (LLC‐WT/K148R), respectively. The cells with lentiviruses were selected using 2–4 µg/mL puromycin.

### Tissue Samples

NSCLC tissues and corresponding para‐carcinoma tissues (20 pairs) for LC‐MS/MS analysis were collected from Tangdu Hospital, with approval from the ethics committees of Tangdu Hospital, Fourth Military Medical University (Project Number: 82203632). The tissue microarrays for IHC staining and multi‐color immunofluorescence staining were obtained from Shanghai Outdo Biotech Co., Ltd. and Shaanxi Avilabio Co., Ltd.

### Plasmids and Recombinant Proteins

The plasmids eGFP‐N1‐CD147 and eGFP‐N1‐CD147‐K148R were constructed in the laboratory. A DNA sequence encoding human CD147 (NM_198589) was inserted into the eGFP‐N1 vector using HindIII/BamHI to construct the eGFP‐N1‐CD147 plasmid, which was then modified to generate a CD147 mutant (eGFP‐N1‐CD147‐K148R) using a site‐directed mutagenesis kit (SDM‐15, SBS Genetech). Some plasmids were obtained from Genscript (pcDNA3.1‐NSD2 (EcoRI/ApaI)) and Tsingke Biotechnology Co.,Ltd. (pRL‐TK, peGFP‐N1‐ZBTB32 (HindIII/SacII)). DNA sequences encoding the CCL5 gene promoter were cloned and inserted into the pGL3‐Basic vector using NheI/XhoI to construct plasmids (pGL3‐Basic‐CCL5 promoter (−1000/−1), pGL3‐Basic‐CCL5 promoter (−500/−1), pGL3‐Basic‐CCL5 promoter (−400/−1), pGL3‐Basic‐CCL5 promoter (−300/−1), pGL3‐Basic‐CCL5 promoter (Del), pGL3‐Basic‐CCL5 promoter (Mut)). Recombinant NSD2 was obtained from Genscript, and recombinant CyPA protein (10436‐H08E) was purchased from Sinobiological. The cells in this study were treated with recombinant CyPA at a concentration of 100 ng mL⁻^1^. Recombinant CD147 and CD147‐K148R proteins were expressed and purified according to previous methods.^[^ ^40^ ^]^

### Chemical Reagents

The p38 MAPK inhibitor (SB203580) was purchased from Abcam (ab120162) and used in cell experiments at a concentration of 10 µM. U‐46619 (HY‐108566, MCE, 10 µM) was utilized as a p38 MAPK activator, while Maraviroc (HY‐13004, MCE, 100 nM) was used as a CCR5 inhibitor in this study.

### LC‐MS/MS

Total protein was extracted from NSCLC tissues and corresponding para‐carcinoma tissues (20 pairs) using RIPA lysis buffer (P0013B, Beyotime) supplemented with protease inhibitor (EDTA‐free, 04693159001, Roche) and PSMF (ST505, Beyotime). Then, the samples were reduced with DL‐dithiothreitol (HY‐15917, MCE), alkylated with iodoacetamide (I1149, Sigma‐Aldrich), and digested with Trypsin/Lys‐C Mix (V5072, Promega). Subsequently, the peptides were purified using Pierce C18 Tips (87784, Thermo Fisher Scientific) and analyzed by LC‐MS/MS on an Orbitrap Eclipse mass spectrometer (Thermo Fisher Scientific) in data‐dependent mode. MS/MS fragmentation of the 20 most intense peaks was collected for every full MS scan. Finally, the data were obtained by searching for human CD147 (NP_940991.1) using Thermo Proteome Discoverer software (Thermo Fisher Scientific).

### Generation of the Anti‐CD147‐K148me2 Antibody

Balb/c mice (female, 7–8 weeks old) were used to generate the anti‐CD147‐K148me2 antibody. The modified peptide K148me2‐KLH (80 µg, QYAOBIO) was injected subcutaneously into the neck and back of mice after being mixed with an equal volume of Freund's complete adjuvant. The mixture of antigen and Freund's incomplete adjuvant was injected in the same way after three weeks. This procedure was repeated once. Two weeks later, the modified peptide K148me2‐KLH (100 µg) was intraperitoneally injected, and the serum antibody titer was measured by ELISAs after one week. The booster immunization was conducted 3 days prior to cell fusion with 100 µg of antigen. SP2/0 cells were fused with spleen cells isolated from mice using PEG1500. The targeted cell line was obtained via HAT and HT medium screening and ELISA detection. The targeted antibody was named 12C8. Finally, the supernatant of monoclonal cells was collected and purified to generate the anti‐CD147‐K148me2 antibody (12C8).

### Gene Silencing and Overexpression

The targeted cells were transfected with the corresponding siRNAs or plasmids using a versatile DNA/siRNA transfection reagent (PT‐114‐15, Polyplus). The detailed procedure was carried out according to the manufacturer's instructions. The sequences of the siRNAs used are listed in Table S5 (Supporting Information).

### Real‐Time PCR (RT‐PCR)

Total RNA was extracted from different samples with a Total RNA Kit II (D6934‐01, OMEGA‐BIO‐TEK) following the manufacturer's instructions. Subsequently, an equal amount of RNA was reverse‐transcribed into cDNA with the PrimeScript RT Master Mix Kit (RR036A, Takara). TB Green Premix Ex Taq II (RR820A, Takara) was used to perform RT‐PCR assays. The sequences of the different primers used are listed in Table S6 (Supporting Information).

### Western Blot and Dot Blot

The samples from cell lines and tissues were lysed with RIPA lysis buffer (P0013B, Beyotime) containing protease inhibitor (EDTA‐free, 04693159001, Roche) and PSMF (ST505, Beyotime). After incubation on ice for 10 min, the protein supernatant was collected and quantified with a Pierce BCA Protein Assay Kit (23227, Thermo Fisher Scientific). The nucleoproteins and cytosolic proteins were extracted with a Nuclear and Cytoplasmic Protein Extraction Kit (P0028, Beyotime). The samples were mixed with SDS‐PAGE Sample Loading Buffer (P0015L, Beyotime) and boiled at 100 °C for 5 min. Subsequently, the samples were loaded on SDS‐PAGE gels and then transferred to PVDF membranes (ISEQ00010, IPVH00010, Millipore). After being blocked with NcmBlot blocking buffer (P30500, NCM Biotech), the membranes were incubated with the corresponding primary antibodies at 4 °C overnight or at room temperature for 2–3 h. The images were developed following incubation with different secondary antibodies at room temperature for 1 h. The antibodies used in the western blot analysis were as follows: anti‐β‐actin from Proteintech (66009‐1‐Ig), dilution 1:5000; anti‐CD147 (HAb18) produced in the laboratory, dilution 1:3000; anti‐CD147‐K148me2 antibody (12C8) produced in the laboratory, dilution 1:2000; anti‐NSD2 from CST (65127s), dilution 1:2000; anti‐α‐Tubulin from CST (3873s), dilution 1:3000; anti‐CCL5 from HUABIO (ET1705‐70), dilution 1:500; anti‐CyPA from HUABIO (ET1703‐33), dilution 1:1000; anti‐JNK from CST (9252s), dilution 1:1000; anti‐p‐JNK from CST (9251s), dilution 1:1000; anti‐p38 from HUABIO (ET1602‐26), dilution 1:2000, anti‐p‐p38 from HUABIO (ER1903‐01), dilution 1:2000; anti‐ERK1/2 from HUABIO (ET1601‐29), dilution 1:2000; anti‐p‐ERK1/2 from CST (4370s), dilution 1:2000; anti‐ZBTB32 from Invitrogen (PA5‐113557), dilution 1:1000; anti‐BSA from Proteintech (66201‐1‐Ig), dilution 1:5000; anti‐GAPDH from Proteintech (60004‐1‐Ig), dilution 1:50000; anti‐Lamin B1 from Abcam (ab16048), dilution 1:5000; goat anti‐mouse IgG (H+L) from Thermo Fisher Scientific (31430), dilution 1:5000; goat anti‐rabbit IgG (H+L) from Thermo Fisher Scientific (31460), dilution 1:5000. NC membranes (HATF00010, Millipore) were used for dot blot assays.

### Structural Modeling of the CD147‐NSD2, CyPA‐CD147‐K148me2 and CyPA‐CD147 Complexes

The crystal structures of the ECD of CD147 (PDB ID: 3B5H),^[^ ^40^ ^]^ the SET domain of NSD2 in the SAM‐bound state (PDB ID: 5LSU),^[^ ^41^ ^]^ and CyPA (PDB ID: 4YUN)^[^ ^42^ ^]^ were retrieved from the Protein Data Bank. For the CD147‐NSD2 complex, K148 of CD147, which is involved in di‐methylation, was set as the attraction restraint, and the initial complex models were built with the ClusPro web server.^[^ ^43^ ^]^ Based on the substrate recognition mechanism the most reasonable model was refined with HADDOCK,^[^ ^44^ ^]^ a hybrid algorithm that combines molecular docking and molecular dynamics simulation. The top‐rated CD147‐NSD2 complex model was used for further analysis. For the CyPA‐CD147 complex, ab initio free docking using HDOCK^[^ ^45^ ^]^ was utilized to generate the initial models. Further analysis revealed that K148 of CD147 and R69 and T107 of CyPA, which were reported to interact with CD147,^[^ ^46^ ^]^ were located at the interface of the top‐rated model. Di‐methylation modification was added to K148 of CD147 in this model using PyTMs, and both the CyPA‐CD147 and CyPA‐CD147‐K148me2 complexes were finally refined by the MD‐based HADDOCK Refinement interface. The free energy of binding (ΔG) of the complex was calculated by PRODIGY,^[^ ^47^ ^]^ and PyMOL^[^ ^48^ ^]^ was used for structural analysis and figure presentation.

### RNA‐seq

Total RNA from CD147‐WT‐ and CD147‐K148R‐overexpressing A549 cells was collected for RNA‐seq, and A549 cells with a mock plasmid were used as a control. The samples were performed by Singleron, and the RNA‐seq data were analyzed by Gene Denovo Biotechnology Co., Ltd.

### Co‐IP Assay

Co‐IP assays were performed to identify the interaction of targeted proteins using a Pierce co‐IP kit (26149, Thermo Fisher Scientific) according to the manufacturer's instructions. The HAb18 antibody was used to capture the CD147 protein, and western blot was employed to detect the proteins that interact with CD147. IgG antibody (SP031, Solarbio) served as a negative control.

### In Vitro Methyltransferase Assay

The recombinant NSD2 protein was co‐incubated with the K148 peptide and recombinant proteins (His‐CD147‐WT and His‐CD147‐K148R) in methyltransferase buffer for 2–3 h at 37 °C according to established methods.^[^ ^20^ ^]^

### ELISAs

ELISAs were conducted to detect the specificity and sensitivity of the 12C8 antibody. Specifically, the modified peptide K148me2 and unmodified peptide K148 were coated on ELISA strips overnight at 4 °C. After blocking with 5% nonfat milk, different concentrations of 12C8 were used to detect the peptide antigen at 37 °C for 1 h. Subsequently, the secondary antibody with HRP (31430, Thermo Fisher Scientific) was added to all wells at 37 °C for 50 min. The data were obtained by measuring the absorbance at 450 nm after adding the substrate solution and stop solution. CCL5 secretion in culture medium and antibody subtype identification of 12C8 were performed using a Human CCL5 ELISA Kit (440807, Biolegend) and a Mouse Monoclonal Antibody Isotype ELISA Kit (EM1812, FineTest), respectively. The procedure was conducted according to the manufacturer's instructions.

### IHC Staining and Analysis

Tissue microarrays of NSCLC (Shanghai Outdo Biotech Co., Ltd.) and tumor tissues from a mouse model were used for IHC staining. The methods for staining and analysis were described previously.^[^ ^20^ ^]^ The primary antibodies for the following proteins were used: anti‐CD147‐K148me2 (12C8, produced in the laboratory, dilution 1:200), anti‐CD147 (HAb18, produced in the laboratory, dilution 1:200), and anti‐Ki‐67 (ab16667, Abcam, dilution 1:200).

### Immunofluorescence Staining

The cells were fixed and permeabilized with 4% paraformaldehyde and 0.2% Triton X‐100, respectively. After blocking with goat serum, an anti‐ZBTB32 antibody (PA5‐113557, Invitrogen, dilution 1:100) was used to detect the ZBTB32 protein overnight at 4 °C. Images were obtained under a confocal florescence microscope following incubation with a secondary antibody (Alexa Fluor 555‐labeled, highly cross‐adsorbed donkey anti‐rabbit IgG (H+L), A‐31572, Thermo Fisher Scientific, dilution 1:200) and DAPI Staining Solution (C1006, Beyotime). For the immunofluorescence staining of NSCLC tissue samples, the sections were dewaxed and retrieved with citrate buffer (pH 6.0). After serum blockade, the sections were incubated with rabbit polyclonal anti‐CD147 antibody (produced in the laboratory, dilution 1:200) and anti‐NSD2 antibody (ab75359, Abcam, dilution 1:200) overnight at 4 °C. Then, the secondary antibodies (Alexa Fluor 488‐labeled, highly cross‐adsorbed donkey anti‐mouse IgG (H+L), A‐21202, Thermo Fisher Scientific, dilution 1:200; Alexa Fluor 555‐labeled, highly cross‐adsorbed donkey anti‐rabbit IgG (H+L), A‐31572, Thermo Fisher Scientific, dilution 1:200) were used to incubate the slides. DAPI Staining Solution (C1006, Beyotime) was used to detect the cell nuclei. For multi‐color immunofluorescence staining, tissue samples were performed using Opal 6‐Plex Manual Detection Kit (AKOYA Biosciences, NEL811001KT) according to the manufacturer's instructions. The following antibodies were used: anti‐CD147‐K148me2 antibody (12C8, produced in the laboratory, dilution 1:200), anti‐CD68 antibody (ab213363, Abcam, dilution 1:1000), anti‐F4/80 antibody (30325s, CST, dilution 1:400), anti‐CD206 antibody (24595s, CST, dilution 1:400).

### Dual‐Luciferase Reporter Assay

HEK293T cells were transfected with plasmids encoding the CCL5 gene promoter, pRL‐TK, and the peGFP‐N1‐ZBTB32 or peGFP‐N1 vector by a versatile DNA/siRNA transfection reagent (PT‐114‐15, Polyplus) for 36–48 h. The relative luciferase activity was determined using a Dual‐Luciferase Reporter Assay System (E1980, Promega) according to the manufacturer's instructions.

### Online Bioinformatics Analysis

The binding site of the transcription factor ZBTB32 to the CCL5 gene promoter was predicted by the JASPAR database. The Tumor Immune Single‐cell Hub 2 (TISCH2) is an scRNA‐seq database focused on the TME that provides detailed information on gene expression at the single‐cell level.^[^ ^49^, ^50^ ^]^ TISCH2 was used to analyze CCR5 levels in different cell subtypes in the TME.^[^ ^51^ ^]^ TIMER is a comprehensive resource for the systematic analysis of immune infiltrates across diverse cancer types.^[^ ^52^, ^53^, ^54^ ^]^ GEPIA is an interactive web server for analyzing RNA sequencing expression data using a standardized processing pipeline.^[^ ^55^ ^]^ TISIDB is a web portal for evaluating tumor and immune system interactions.^[^ ^56^ ^]^ The TIMER, GEPIA, and TISIDB databases were used to analyze the correlation between ZBTB32 and CCL5, and the TISIDB database was also used to assess the correlation between ZBTB32 or CCL5 expression and macrophage infiltration in NSCLC.

### CCK‐8 Assay

The cell proliferation assay was conducted using a CCK‐8 kit (C0005, Topscience). The tumor cells (2 000 cells per well) were seeded into 96‐well plates and measured with CCK‐8 reagent at different time points (0, 24, 48, 72 h).

### Cell Wound Healing Assay

The tumor cells were seeded into 6‐well plates with 10% FBS in RPMI‐1640 medium and cultured at 37 °C and 5% CO_2_ overnight. Then, scratches were created by the tips of the pipettes, and the medium was replaced with 2% FBS RPMI‐1640 medium. Images were obtained at fixed sites (0 and 24 h).

### Cell Chemotaxis Assay

THP‐1 cells were induced to differentiate into M0 macrophages by PMA (P1585, Sigma‐Aldrich, 100 ng mL⁻^1^) for 48 h and then treated with IL‐4 (204‐GMP‐050, R&D Systems, 20 ng mL⁻^1^) and IL‐13 (200‐13‐10UG, Peprotech, 20 ng mL⁻^1^) to obtain M2‐like macrophages for 48 h.^[^ ^57^ ^]^ The expression of CD11b (101206, Biolegend), CD163 (326510, Biolegend), and CD206 (321106, Biolegend) in THP‐1‐derived M2 macrophages (TDMMs) was identified using flow cytometry. Cell chemotaxis assays were performed using Millicell Cell Culture Inserts & Plates (PTEP24H48, Millipore, pore size 8 µm). Briefly, TDMM cells (5 × 10^3^ cells/well) and NSCLC cells (3 × 10^4^ cells/well) were plated in the upper chamber and lower chamber, respectively, at 37 °C in a 5% CO_2_ incubator for 24 h. Some co‐culture systems were treated with an anti‐CCL5 antibody (ET1705‐70, HUABIO, 5 µg mL⁻^1^), recombinant CyPA protein (10436‐H08E, Sinobiological, 100 ng mL⁻^1^), and Maraviroc (HY‐13004, MCE, 100 nM). The TDMM cells in the upper chamber were stained with 0.5% crystal violet stain solution (60506ES60, Yeasen Biotech) for 20 min, and the cells in the interior of the upper chamber were removed with a cotton swab. Images of three fields in one group were obtained under a microscope, and all experiments were independently repeated three times.

### In Vivo Mouse Experiments

The mouse experiments were approved by the Institutional Animal Care and Use Committee of the National Translational Science Center for Molecular Medicine (2022‐NTSCMM‐ID008). The mice (C57BL/6J, female, 6–8 weeks old, Beijing HFK Bioscience Co., Ltd.) were injected subcutaneously with LLC‐WT (2 × 10^6^ cells/mouse, n = 6) or LLC‐K148R cells (2 × 10^6^ cells/mouse, n = 6). The body weight and tumor volume were measured every two days. At the experimental endpoint, the tumor masses were collected to extract total proteins, which were used to assess the expression of ZBTB32 and CCL5. The remaining tissues were fixed, embedded, and sliced to identify Ki‐67 expression and F4/80+CD206+ cell infiltration in tumor tissues. For analysis of the role of 12C8 in tumor treatment, an in vivo test was performed by administering 12C8 (3 mg kg^−1^) intravenously three times, with an equal amount of IgG used as a control.

### Statistical Analysis

The data in this study were analyzed using GraphPad Prism. Unpaired Student's t test and unpaired t test with Welch's correction with a two‐tailed distribution were used to determine the statistical significance. Dot plots were used to present the data from at least three independent experiments (mean±SEM). Spearman correlation analysis was performed to assess the association between ZBTB32 expression and CCL5 expression or macrophage infiltration, as well as the correlation between CD147‐K148me2 levels and M2‐like TAM infiltration. The overall survival of NSCLC patients was calculated using Kaplan‐Meier analysis and the log‐rank test. The associations between CD147‐K148me2 or CD147 levels and different clinicopathological parameters in NSCLC patients were analyzed using the Chi‐square test or Fisher's exact test. The body weight and tumor volume of the mice and the 12C8 binding ability for the peptide K148me2 were analyzed by two‐way ANOVA. A P value less than 0.05 was considered to indicate statistical significance.

## Conflict of Interest

The authors declare no conflict of interest.

## Author Contributions

K. W., X. C., P. L., J. W., and Q. H. contributed equally to this work. K. W. was responsible for data acquisition, analysis, investigation, and writing original draft; X. C., P. L., J. W., and Q. H. were responsible for data acquisition and analysis; Z.N. C., J. T., H. W., Y. T., M. S., M. Q., B. H., Y. Z., L. L., and R. Y. provided the methods, resources, and advice for the study; L. C., R. C., P. Z., and H. B. were responsible for the supervision, conceptualization and design of the study.

## Supporting information

Supporting Information

## Data Availability

The data that support the findings of this study are available from the corresponding author upon reasonable request.
