# Supplementary material for: CD147‐K148me2‐Driven Tumor Cell‐Macrophage Crosstalk Provokes NSCLC Immunosuppression via the CCL5/CCR5 Axis
Source: Adv Sci (Weinh). 2024 Jun 14;11(29):2400611. doi: 10.1002/advs.202400611 (PMC11304266; doi:10.1002/advs.202400611)
Supplement: Supplementary file 1 — Supporting Information [file ADVS-11-2400611-s001.docx]

Supporting Information

**CD147-K148me2-Driven Tumor Cell-Macrophage Crosstalk Provokes NSCLC Immunosuppression via the CCL5/CCR5 Axis**

*Ke Wang, Xiaohong Chen, Peng Lin, Jiao Wu, Qiang Huang, Zhi-Nan Chen, Jiale Tian, Hao Wang, Ye Tian, Mingyan Shi, Meirui Qian, Bengang Hui, Yumeng Zhu,* *Ling Li, Rui Yao, Huijie Bian*, Ping Zhu*, Ruo Chen*, Liang Chen**


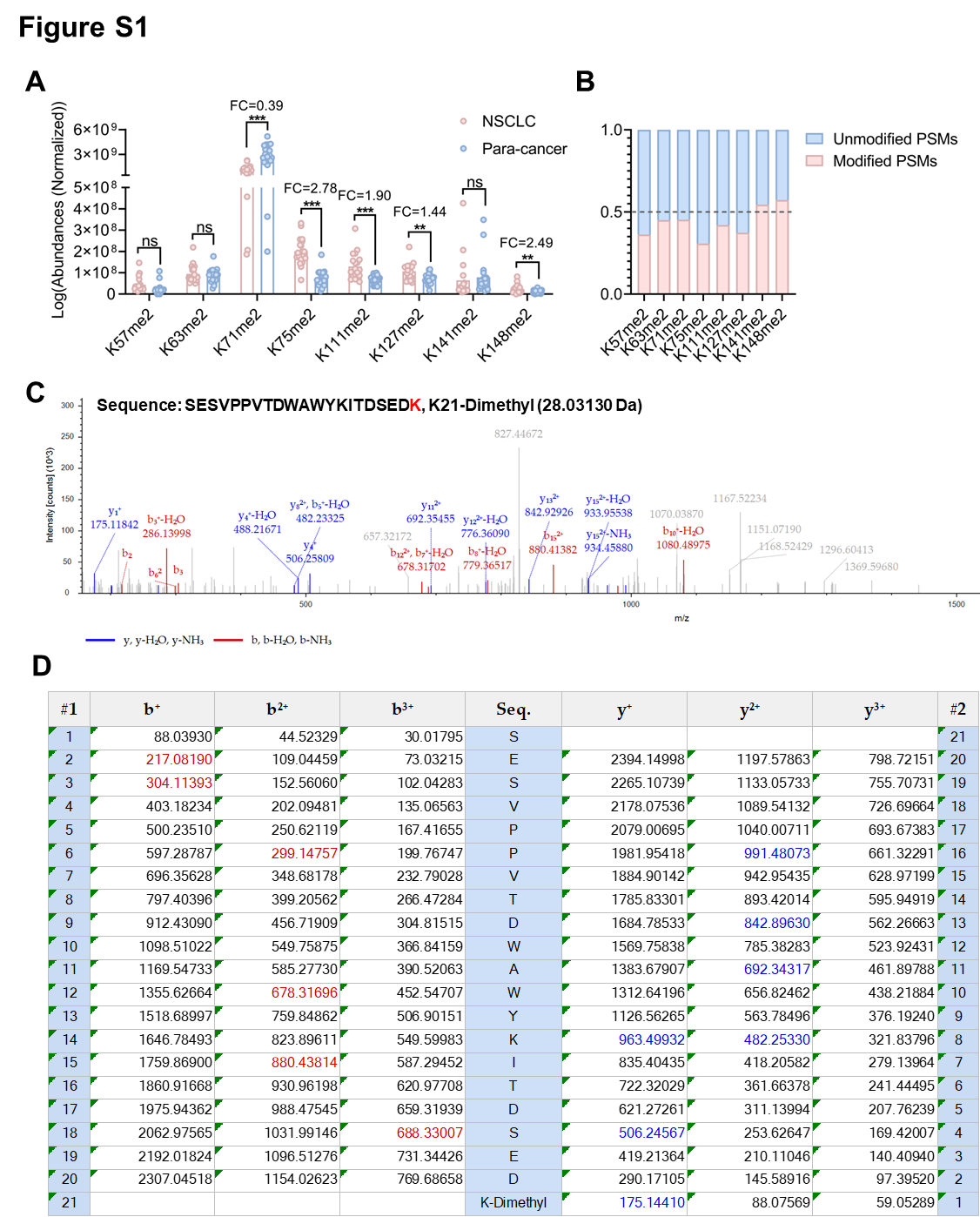


**Figure S1.** CD147 di-methylation at Lys148 is increased in NSCLC patients. A) The di-methylations of CD147-ECD at different lysine sites were detected in 20 paired NSCLC tissues and their corresponding para-carcinoma tissues using LC-MS/MS (**P<0.01; ***P<0.001; ns, not significant; FC, fold change). B) The ratio of modified PSMs to unmodified PSMs for the peptide CD147 was analyzed by LC-MS/MS. C, D) The LC-MS/MS spectrum (C) and ion series (D) of the peptide SESVPPVTDWAWYKITDSEDK, which has a mass of +28.03130 Da at residue Lys21.


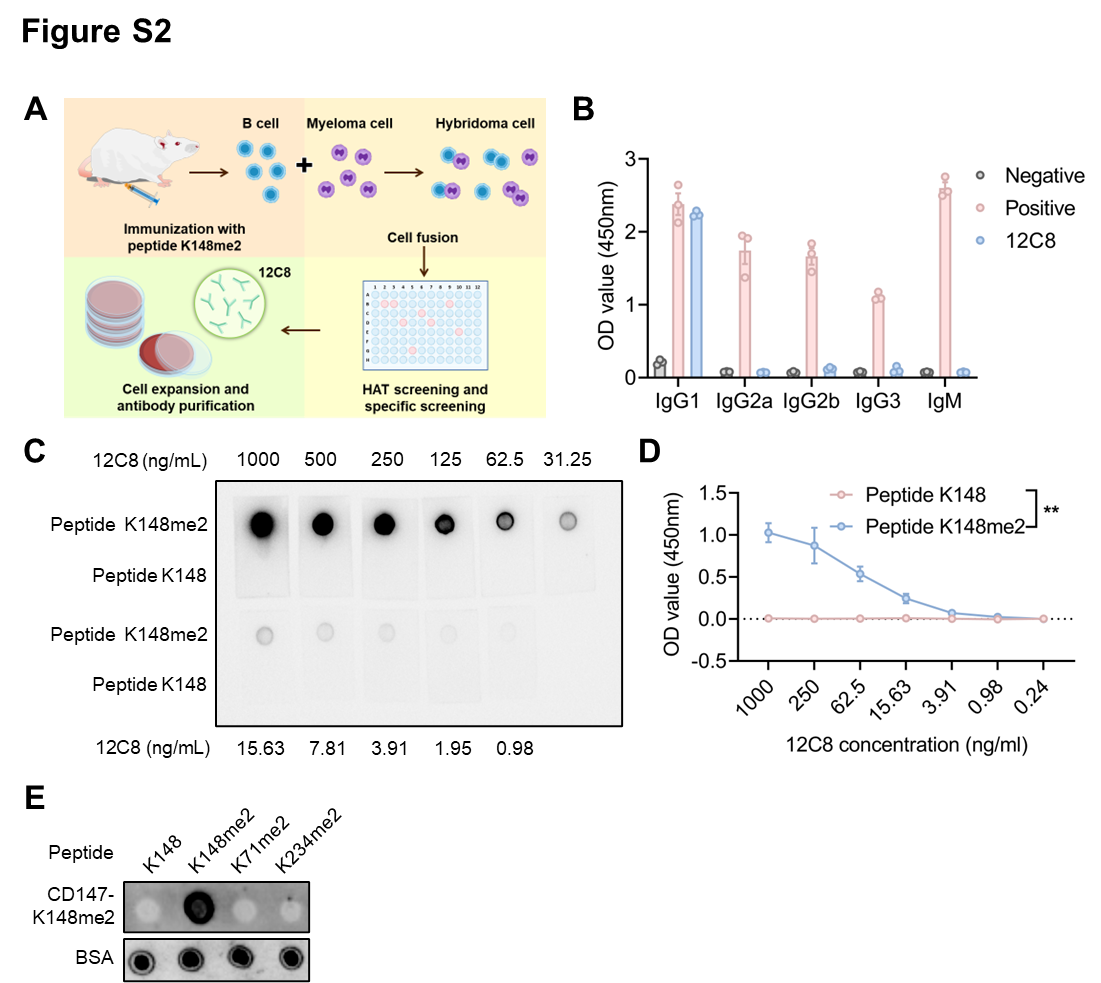


**Figure S2.** Generation and identification of an anti-CD147-K148me2 antibody (12C8). A) Schematic diagram of the generation of an anti-CD147-K148me2 antibody (12C8). B) Antibody subtype identification of 12C8 was performed by ELISAs. C-E) The specificity of 12C8 was verified by dot blot (C, E) and ELISAs (**P<0.01) (D). K148, K71me2, K148me2, and K234me2 are synthetic peptides of CD147.


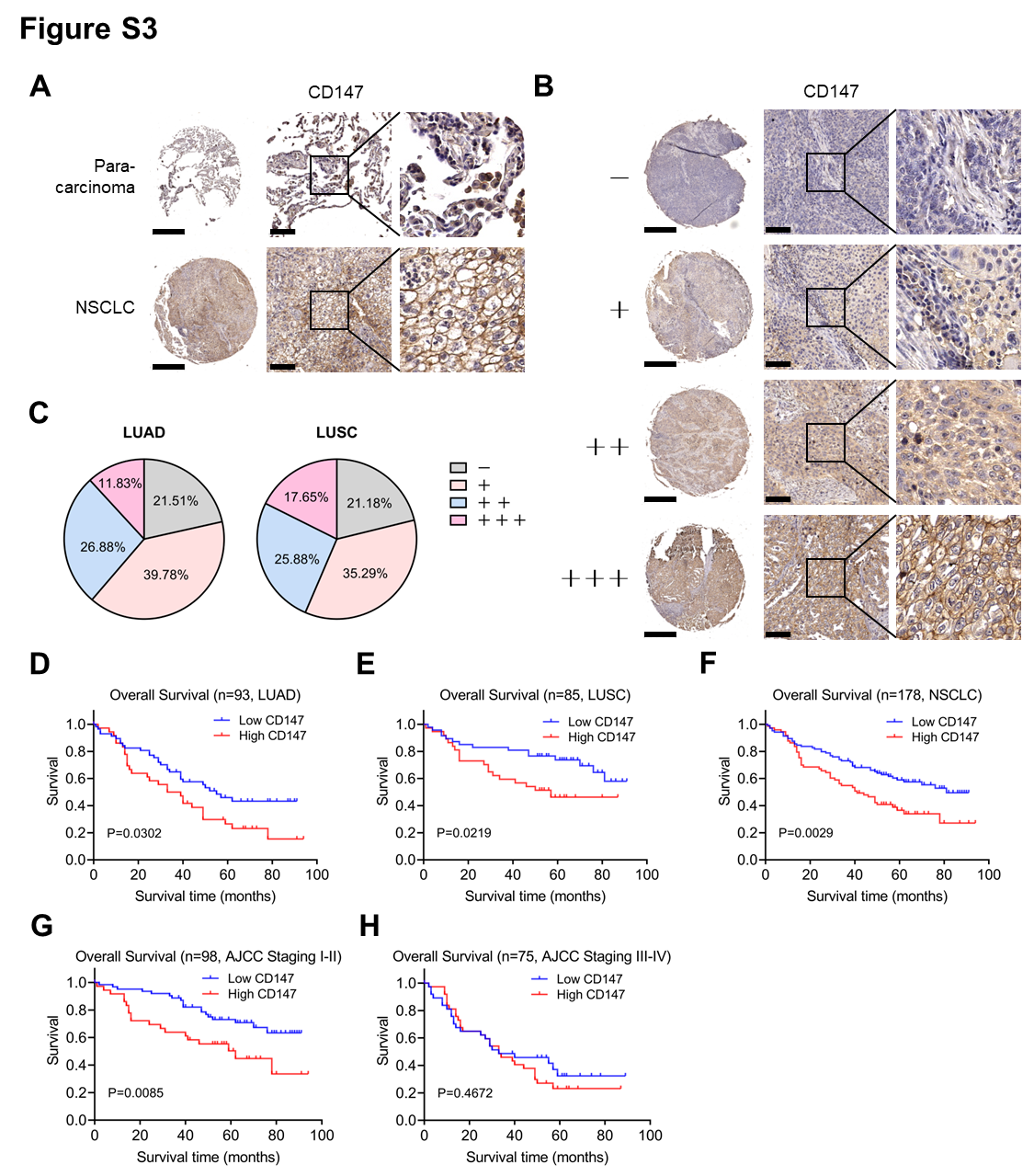


**Figure S3.** CD147 expression is closely associated with the prognosis of NSCLC patients. A) The expression of CD147 in NSCLC tissues and their corresponding para-carcinoma tissues, scale bar, 500 μm (left) and 100 μm (middle). B) Different levels (–, +, ++, and +++) of CD147 expression in NSCLC tissues, scale bar, 500 μm (left) and 100 μm (middle). C) The percentage of patients with positive CD147 expression in the LUAD and LUSC cohorts. D-F) Overall survival of patients with high and low CD147 expression in the LUAD (P=0.0302) (D), LUSC (P=0.0219) (E), and NSCLC (P=0.0029) (F) cohorts. G, H) Overall survival of NSCLC patients with high and low CD147 expression in the early NSCLC (P=0.0085) (G) and advanced NSCLC (P=0.4672) (H) cohorts.


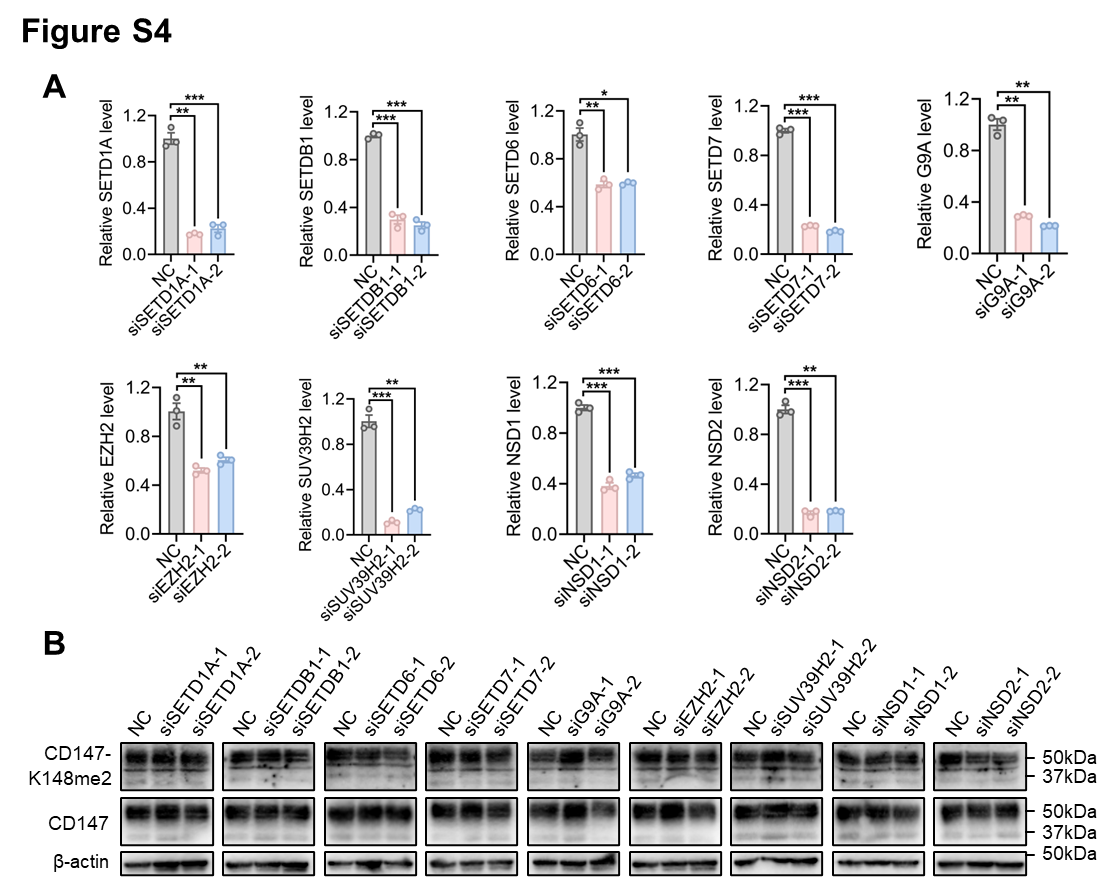


**Figure S4.** The screening of methyltransferase for CD147-K148me2. A) The silencing of different methyltransferases (SETD1A, SETDB1, SETD6, SETD7, G9A, EZH2, SUV39H2, NSD1, and NSD2) in H460 cells was verified by RT-PCR (*P<0.05; **P<0.01; ***P<0.001; n=3). B) The levels of CD147-K148me2 and CD147 were determined in H460 cells with different methyltransferases knocked down (SETD1A, SETDB1, SETD6, SETD7, G9A, EZH2, SUV39H2, NSD1, and NSD2) by western blot.


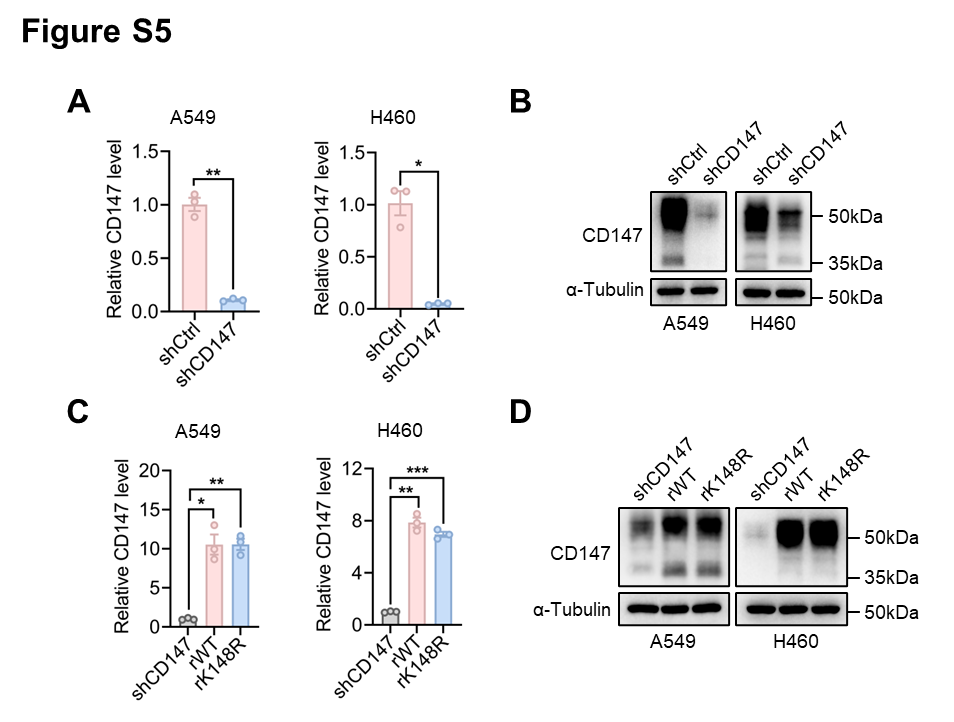


**Figure S5.** Identification of cell lines generated by lentiviral infection. A, B) CD147 expression was determined in A549/H460-shCD147 cell lines by RT-PCR (*P<0.05; **P<0.01; n=3) (A) and western blot (B). C, D) The expression of CD147 was determined in rWT and rK148R cells (A549 and H460) by RT-PCR (*P<0.05; **P<0.01; ***P<0.001; n=3) (C) and western blot (D).


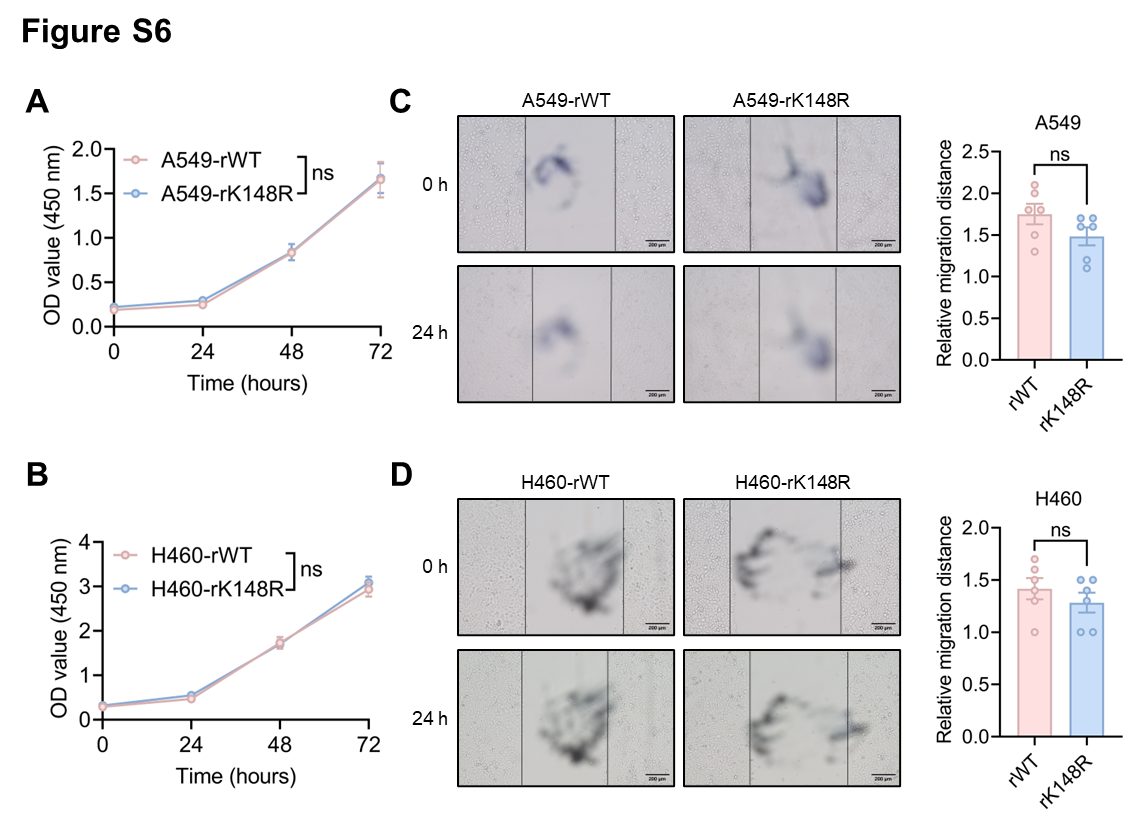


**Figure S6.** CD147-K148me2 has no impact on the proliferation or migration of NSCLC cells. A, B) The proliferation of rWT and rK148R cells (A549 (A) and H460 (B)) was analyzed using a CCK-8 assay (ns, not significant). C, D) The migration of rWT and rK148R cells (A549 (C) and H460 (D)) was analyzed by cell wound healing assay, scale bar, 200 μm (ns, not significant; n=6), the black background on the picture is a marker of the fixed sites.


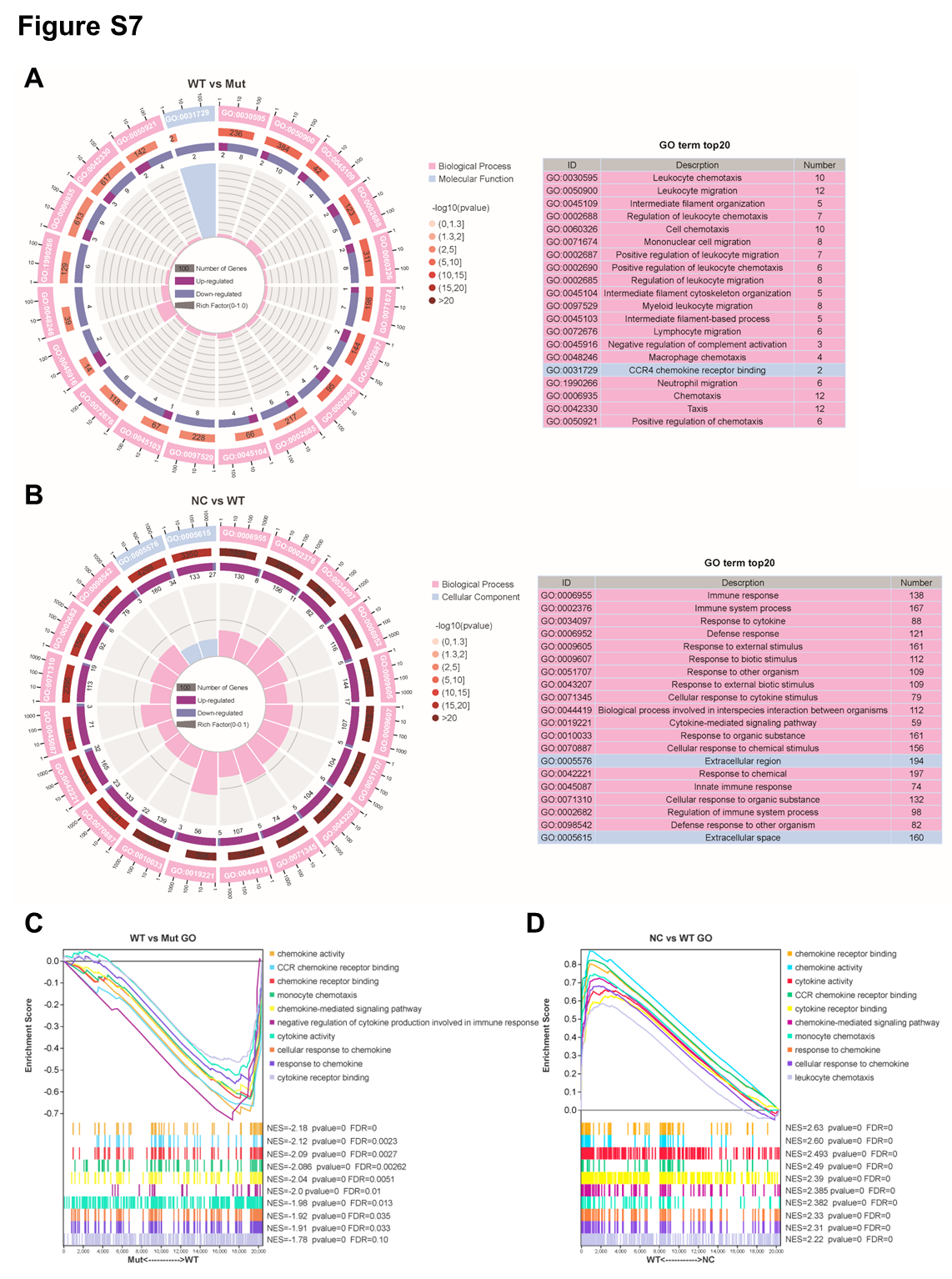


**Figure S7.** GO analysis and GSEA using RNA-seq data. A, B) The top 20 GO terms of DEGs in the WT vs. Mut (A) and NC vs. WT (B) groups were shown. C, D) GSEA was conducted in the WT vs. Mut (C) and NC vs. WT (D) groups to identify the differences of a set of genes in specific GO terms, NES, normalized enrichment score.


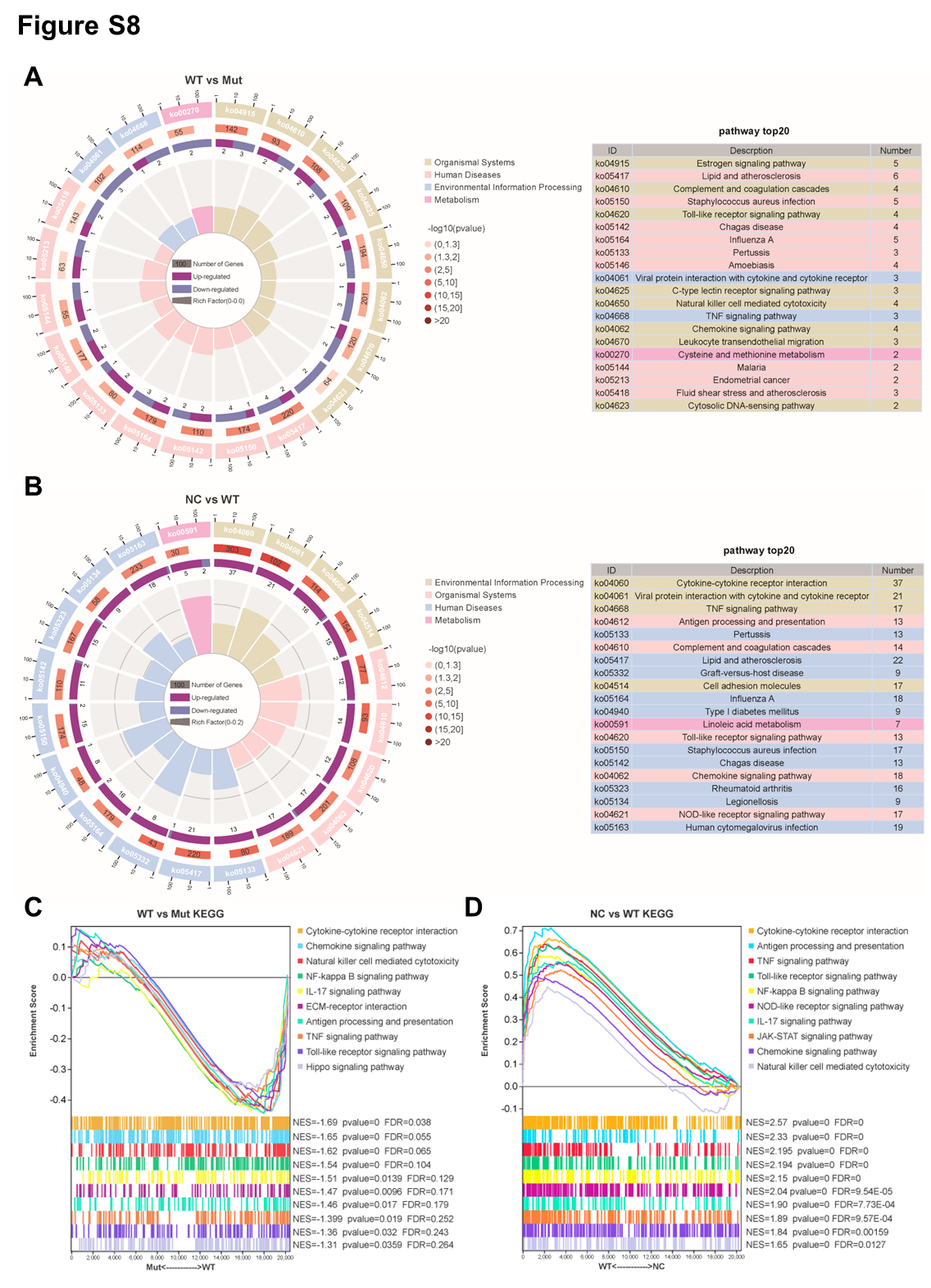


**Figure S8.** KEGG analysis and GSEA using RNA-seq data. A, B) The top 20 KEGG pathways of DEGs in the WT vs. Mut (A) and NC vs. WT (B) groups were shown. C, D) GSEA was conducted in the WT vs. Mut (C) and NC vs. WT (D) groups to identify the differences of a set of genes in specific KEGG pathway, NES, normalized enrichment score.


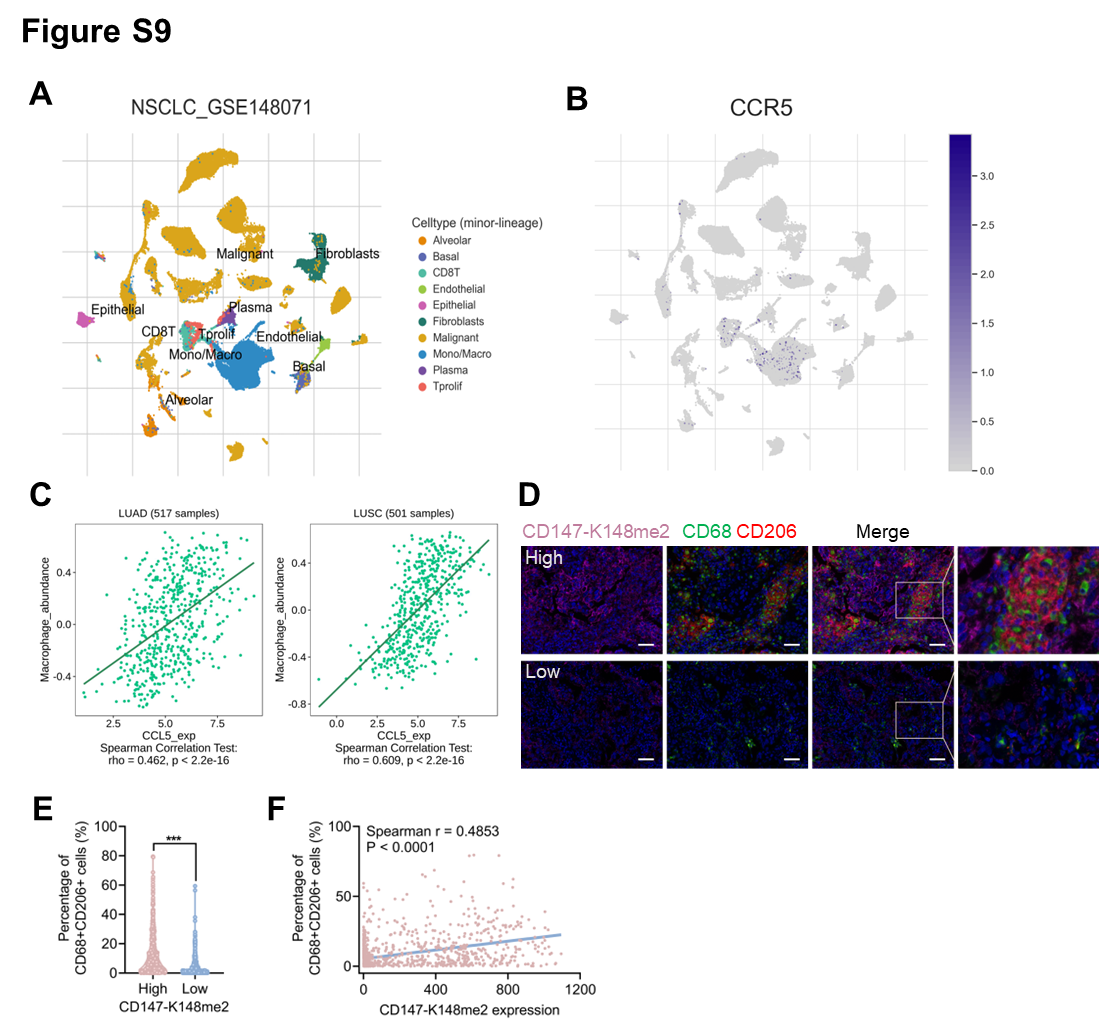


**Figure S9.** The level of CD147-K148me2 is closely associated with M2-like TAM infiltration in NSCLC tissues. A, B) The TISCH2 data were used to analyze the CCR5 levels in different cell subtypes in the TME. C) Data from the TISIDB database were used to analyze the correlation between CCL5 expression and macrophage infiltration in LUAD (R=0.462, P<2.2e-16) and LUSC (R=0.609, P<2.2e-16). D) The expression of CD147-K148me2 (purple) and CD68+CD206+ cell infiltration (CD68, green; CD206, red) were determined in NSCLC tissues by multi-color immunofluorescence staining, scale bar, 50 μm. E) The percentage of CD68+CD206+ cells in NSCLC tissues was compared between the groups with high and low levels of CD147-K148me2 (***P<0.001). F) The correlation between CD147-K148me2 expression and CD68+CD206+ cell infiltration in NSCLC tissues was analyzed using the Spearman correlation test (Spearman r=0.4853, P<0.0001).


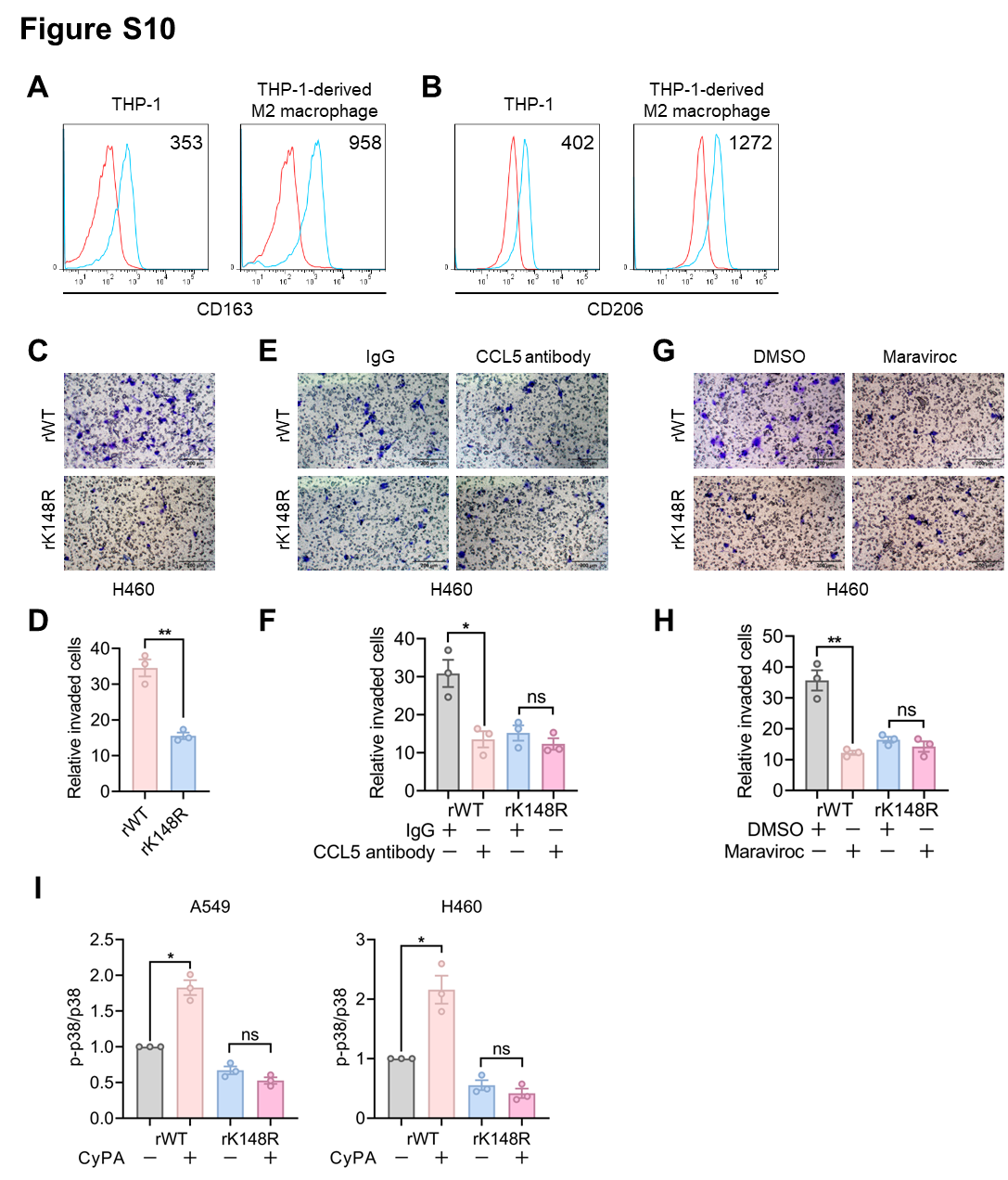


**Figure S10.** CD147-K148me2 promotes M2-like TAM migration in NSCLC. A, B) The expression of CD163 (A) and CD206 (B) was determined in THP-1-derived M2 macrophages using flow cytometry. C, D) A cell chemotaxis assay was conducted by co-incubation of M2-like macrophages and H460-rWT or H460-rK148R cells, scale bar, 200 μm (C), and the quantitative analysis was conducted via three independent experiments (**P<0.01) (D). E, F) A cell chemotaxis assay was conducted by co-incubation of M2-like macrophages and H460-rWT or H460-rK148R cells with IgG or CCL5 antibody, scale bar, 200 μm (E), and the quantitative analysis was conducted via three independent experiments (*P<0.05; ns, not significant) (F). G, H) A cell chemotaxis assay was conducted by co-incubation of M2-like macrophages and H460-rWT or H460-rK148R cells with DMSO or Maraviroc, scale bar, 200 μm (G), and the quantitative analysis was conducted via three independent experiments (**P<0.01; ns, not significant) (H). I) The quantification of p-p38/p38 in Figure 4G was conducted using three independent experiments (*P<0.05; ns, not significant).


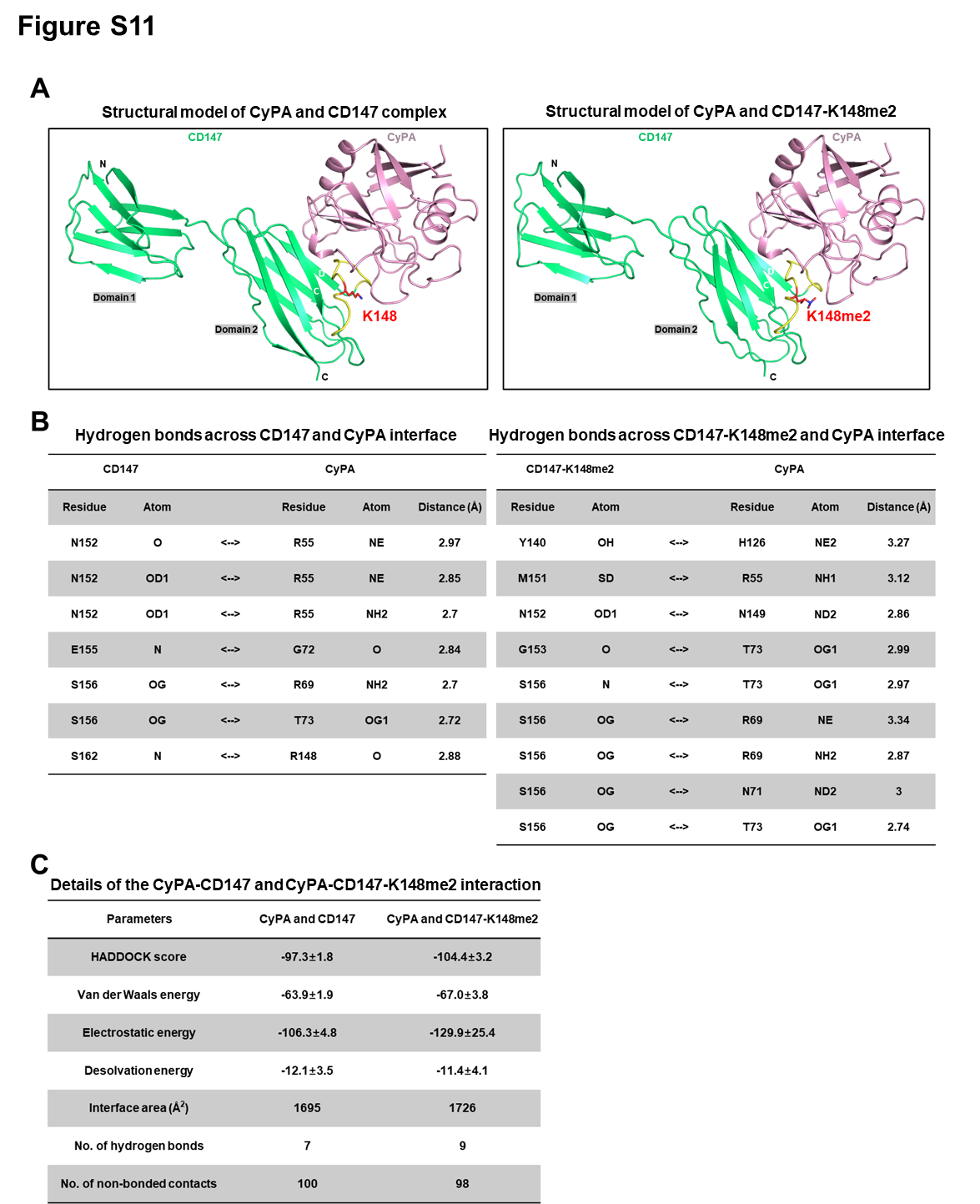


**Figure S11.** Molecular docking of the CyPA-CD147 and CyPA-CD147-K148me2 complexes. A) Models showing the interaction between CD147 (with or without K148me2) and CyPA. The flexible loop that connects β strands C and D of CD147, which is involved in the interaction with CyPA, is highlighted in yellow. B) Hydrogen bonds across the CD147/CD147-K148me2 and CyPA interfaces. C) Details of the CyPA-CD147 and CyPA-CD147-K148me2 interaction.


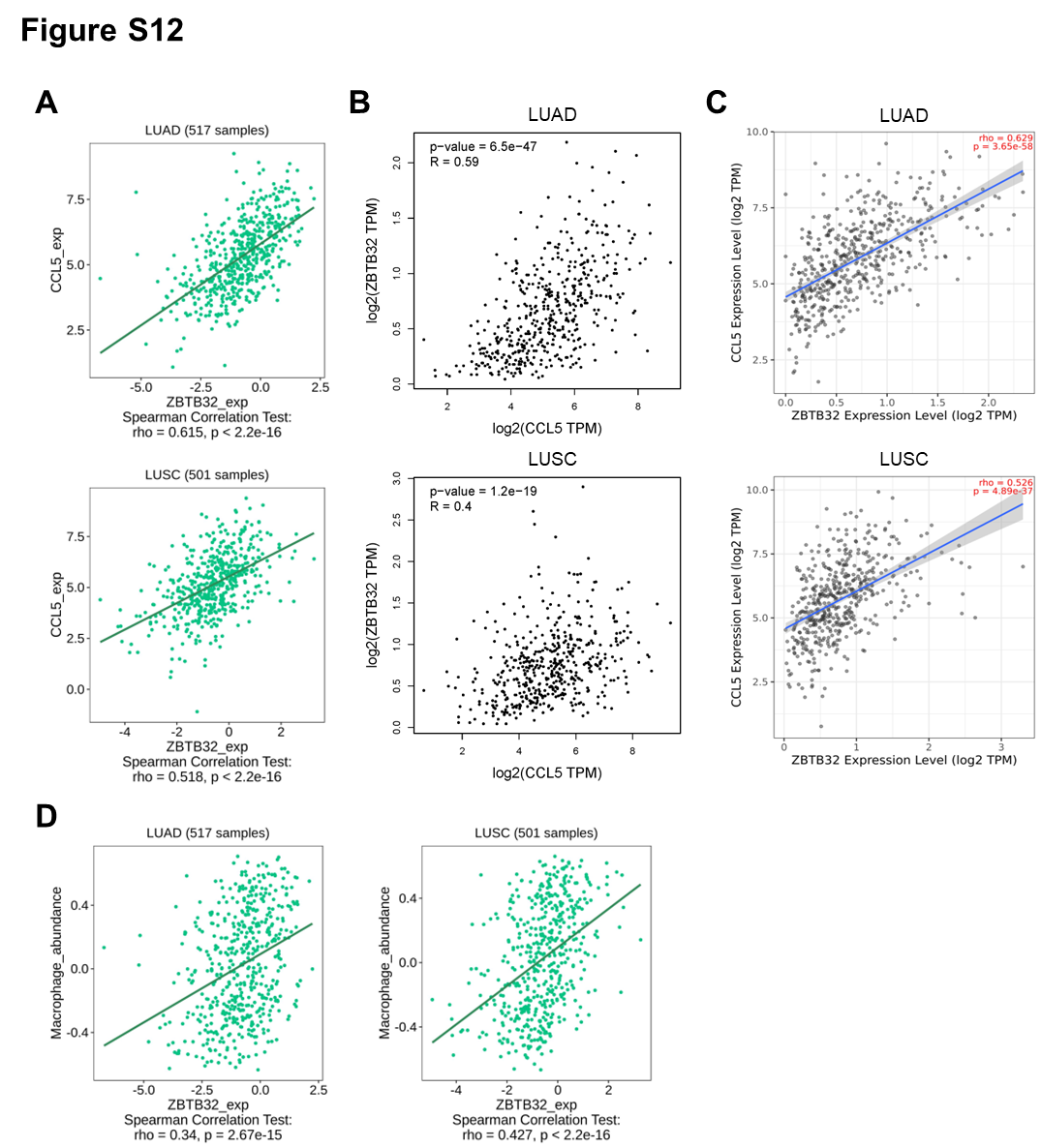


**Figure S12.** Online bioinformatics analysis. A-C) The positive correlation between CCL5 and ZBTB32 expression in NSCLC was analyzed using the TISIDB database (R=0.615, P<2.2e-16, LUAD; R=0.518, P<2.2e-16, LUSC) (A), GEPIA database (R=0.59, P=6.5e-47, LUAD; R=0.4, P=1.2e-19, LUSC) (B), and TIMER database (R=0.629, P=3.65e-58, LUAD; R=0.526, P=4.89e-37, LUSC) (C). D) Data from the TISIDB database were used to analyze the correlation between ZBTB32 expression and macrophage infiltration in LUAD (R=0.34, P=2.67e-15) and LUSC (R=0.427, P<2.2e-16).

**Table S1.** Correlation between different expression levels of CD147-K148me2 with clinicopathological parameters of individuals with LUAD.

| **Parameters** | **N (98)** | **Low CD147-**  **K148me2 (60)** | **High CD147-**  **K148me2 (38)** | **P** |
| --- | --- | --- | --- | --- |
| **Gender** |  |  |  |  |
| Male | 59 | 31 | 28 | **0.03003** |
| Female | 39 | 29 | 10 |  |
| **Age (years)** |  |  |  |  |
| ≥60 | 53 | 33 | 20 | 0.81868 |
| ＜60 | 45 | 27 | 18 |  |
| **Tumor size (cm)** |  |  |  |  |
| ≤3 | 35 | 26 | 9 | **0.04793** |
| ＞3 | 63 | 34 | 29 |  |
| **T staging** |  |  |  |  |
| T1+T2 | 71 | 47 | 24 | 0.05259 |
| T3+T4 | 23 | 10 | 13 |  |
| **N staging** |  |  |  |  |
| N0 | 46 | 31 | 15 | 0.28644 |
| N1+N2+N3+Nx | 51 | 29 | 22 |  |
| **AJCC staging** |  |  |  |  |
| I-II | 54 | 38 | 16 | 0.05303 |
| III-IV | 43 | 22 | 21 |  |
| **Pathological staging** |  |  |  |  |
| I+I-II+II | 61 | 42 | 19 | **0.0466** |
| II-III+III | 37 | 18 | 19 |  |

Cases of ‘‘–’’ and ‘‘+’’ were assigned to the group with low expression levels, whereas cases of ‘‘++’’ and ‘‘+++’’ were assigned to the group with high expression levels. The missing information is listed as follows: T staging (4), N staging (1), AJCC staging (1). The clinical staging of the individuals was based on the AJCC staging (7th edition).

**Table S2.** Correlation between different expression levels of CD147-K148me2 with clinicopathological parameters of individuals with LUSC.

| **Parameters** | **N (85)** | **Low CD147-**  **K148me2 (49)** | **High CD147-**  **K148me2 (36)** | **P** |
| --- | --- | --- | --- | --- |
| **Gender** |  |  |  |  |
| Male | 81 | 45 | 36 | 0.13373 |
| Female | 4 | 4 | 0 |  |
| **Age (years)** |  |  |  |  |
| ≥63 | 45 | 23 | 22 | 0.17729 |
| ＜63 | 38 | 25 | 13 |  |
| **Tumor size (cm)** |  |  |  |  |
| ≤3 | 16 | 13 | 3 | 0.07839 |
| ＞3 | 64 | 34 | 30 |  |
| **T staging** |  |  |  |  |
| T1+T2 | 60 | 36 | 24 | 0.33248 |
| T3+T4 | 19 | 9 | 10 |  |
| **N staging** |  |  |  |  |
| N0 | 46 | 29 | 17 | 0.29406 |
| N1+N2+Nx | 35 | 18 | 17 |  |
| **AJCC staging** |  |  |  |  |
| I-II | 48 | 29 | 19 | 0.2883 |
| III-IV | 33 | 16 | 17 |  |
| **Pathological staging** |  |  |  |  |
| I-II+II | 60 | 35 | 25 | 0.84275 |
| I-III+II-III+III | 25 | 14 | 11 |  |

Cases of ‘‘–’’ and ‘‘+’’ were assigned to the group with low expression levels, whereas cases of ‘‘++’’ and ‘‘+++’’ were assigned to the group with high expression levels. The missing information is listed as follows: Age (2), Tumor size (5), T staging (6), N staging (4), AJCC staging (4). The clinical staging of the individuals was based on the AJCC staging (7th edition).

**Table S3.** Correlation between different expression levels of CD147 with clinicopathological parameters of individuals with LUAD.

| **Parameters** | **N (93)** | **Low CD147**  **(57)** | **High CD147**  **(36)** | **P** |
| --- | --- | --- | --- | --- |
| **Gender** |  |  |  |  |
| Male | 55 | 37 | 18 | 0.15417 |
| Female | 38 | 20 | 18 |  |
| **Age (years)** |  |  |  |  |
| ≥60 | 49 | 29 | 20 | 0.65983 |
| ＜60 | 44 | 28 | 16 |  |
| **Tumor size (cm)** |  |  |  |  |
| ≤3 | 33 | 23 | 10 | 0.21707 |
| ＞3 | 60 | 34 | 26 |  |
| **T staging** |  |  |  |  |
| T1+T2 | 66 | 41 | 25 | 0.63591 |
| T3+T4 | 23 | 13 | 10 |  |
| **N staging** |  |  |  |  |
| N0 | 43 | 28 | 15 | 0.4343 |
| N1+N2+N3+Nx | 49 | 28 | 21 |  |
| **AJCC staging** |  |  |  |  |
| I-II | 50 | 32 | 18 | 0.50204 |
| III-IV | 42 | 24 | 18 |  |
| **Pathological staging** |  |  |  |  |
| I+I-II+II | 60 | 37 | 23 | 0.91997 |
| II-III+III | 33 | 20 | 13 |  |

Cases of ‘‘–’’ and ‘‘+’’ were assigned to the group with low expression levels, whereas cases of ‘‘++’’ and ‘‘+++’’ were assigned to the group with high expression levels. The missing information is listed as follows: T staging (4), N staging (1), AJCC staging (1). The clinical staging of the individuals was based on the AJCC staging (7th edition).

**Table S4.** Correlation between different expression levels of CD147 with clinicopathological parameters of individuals with LUSC.

| **Parameters** | **N (85)** | **Low CD147**  **(48)** | **High CD147**  **(37)** | **P** |
| --- | --- | --- | --- | --- |
| **Gender** |  |  |  |  |
| Male | 81 | 44 | 37 | 0.12872 |
| Female | 4 | 4 | 0 |  |
| **Age (years)** |  |  |  |  |
| ≥63 | 45 | 23 | 22 | 0.26988 |
| ＜63 | 38 | 24 | 14 |  |
| **Tumor size (cm)** |  |  |  |  |
| ≤3 | 16 | 13 | 3 | **0.04861** |
| ＞3 | 64 | 32 | 32 |  |
| **T staging** |  |  |  |  |
| T1+T2 | 60 | 32 | 28 | 0.7279 |
| T3+T4 | 19 | 11 | 8 |  |
| **N staging** |  |  |  |  |
| N0 | 46 | 33 | 13 | **0.00185** |
| N1+N2+Nx | 35 | 13 | 22 |  |
| **AJCC staging** |  |  |  |  |
| I-II | 48 | 30 | 18 | 0.07471 |
| III-IV | 33 | 14 | 19 |  |
| **Pathological staging** |  |  |  |  |
| I-II+II | 60 | 35 | 25 | 0.59153 |
| I-III+II-III+III | 25 | 13 | 12 |  |

Cases of ‘‘–’’ and ‘‘+’’ were assigned to the group with low expression levels, whereas cases of ‘‘++’’ and ‘‘+++’’ were assigned to the group with high expression levels. The missing information is listed as follows: Age (2), Tumor size (5), T staging (6), N staging (4), AJCC staging (4). The clinical staging of the individuals was based on the AJCC staging (7th edition).

**Table S5.** The sequences for siRNAs.

| **siRNA** | **Sense (5'-3')** | **Antisense (5'-3')** |
| --- | --- | --- |
| siCtrl | UUCUCCGAACGUGUCACGUTT | ACGUGACACGUUCGGAGAATT |
| siSETD1A-1 | GUCCCUUCCUCUUGGUUAUTT | AUAACCAAGAGGAAGGGACTT |
| siSETD1A-2 | CGCACAUCUAUGACUUUGUTT | ACAAAGUCAUAGAUGUGCGTT |
| siSETDB1-1 | GGCCUACAGAAAUAAUUGATT | UCAAUUAUUUCUGUAGGCCTT |
| siSETDB1-2 | GGGCUUUCAUGUUAUCUAUTT | AUAGAUAACAUGAAAGCCCTT |
| siSETD6-1 | GCCCUUGUGAUGGCCUAUATT | UAUAGGCCAUCACAAGGGCTT |
| siSETD6-2 | GCCCAUUCCUAAAGGCCAUTT | AUGGCCUUUAGGAAUGGGCTT |
| siSETD7-1 | GGGCAGUAUAAAGAUAACATT | UGUUAUCUUUAUACUGCCCTT |
| siSETD7-2 | GGACCUAAUACUGUUAUGUTT | ACAUAACAGUAUUAGGUCCTT |
| siG9A-1 | GGUGUCCAAUGACACAUCUTT | AGAUGUGUCAUUGGACACCTT |
| siG9A-2 | GUGGCUGUGUCUAUAGCAATT | UUGCUAUAGACACAGCCACTT |
| siEZH2-1 | GCAACACCCAACACUUAUATT | UAUAAGUGUUGGGUGUUGCTT |
| siEZH2-2 | CGGCUUCCCAAUAACAGUATT | UACUGUUAUUGGGAAGCCGTT |
| siSUV39H2-1 | CUUGCCUAGUUUCACUUGATT | UCAAGUGAAACUAGGCAAGTT |
| siSUV39H2-2 | GGUCCUGAUUGUCCCAAUATT | UAUUGGGACAAUCAGGACCTT |
| siNSD1-1 | GGGCCACAUACAAUUUGAATT | UUCAAAUUGUAUGUGGCCCTT |
| siNSD1-2 | GGGCCACUUAACAAGUGAATT | UUCACUUGUUAAGUGGCCCTT |
| siNSD2-1 | GGGCAUUGUUCAAGCAGAATT | UUCUGCUUGAACAAUGCCCTT |
| siNSD2-2 | GCCAGAACAAGCUCUUACATT | UGUAAGAGCUUGUUCUGGCTT |
| siZBTB32-1 | GCAGGAACAGACCAGGUCATT | UGACCUGGUCUGUUCCUGCTT |
| siZBTB32-2 | GGAGAUGGAAGAGUCUGAUTT | AUCAGACUCUUCCAUCUCCTT |

**Table S6.** The sequences for primers.

| **Primers** | **Sequences (5'-3')** |
| --- | --- |
| Human-actin-Forward | CACCATTGGCAATGAGCGGTTC |
| Human-actin-Reverse | AGGTCTTTGCGGATGTCCACGT |
| Human-SETD1A-Forward | TCTAGATCGTCGTGGCGAAG |
| Human-SETD1A-Reverse | GGTCTGCATTCGCACTTTCG |
| Human-SETDB1-Forward | GCTTGCCCAGTCACGGAA |
| Human-SETDB1-Reverse | GCTGTGGAGCCTAAAGGTGAT |
| Human-SETD6-Forward | ACTTGCCTTGGACACAGCTT |
| Human-SETD6-Reverse | TTCCCAGCCCTTTGGTTTGT |
| Human-SETD7-Forward | CACCTGGACGATGACGGATT |
| Human-SETD7-Reverse | CTCCGTCTACATACGTGCCC |
| Human-G9A-Forward | CTGACAACGAGGAGAACATCTG |
| Human-G9A-Reverse | AACAGCACGCAGTCATGGTA |
| Human-EZH2-Forward | AATCAGAGTACATGCGACTGAGA |
| Human-EZH2-Reverse | GCTGTATCCTTCGCTGTTTCC |
| Human-SUV39H2-Forward | GTGCCTTGCCTAGTTTCACT |
| Human-SUV39H2-Reverse | AAGCAGTAACGGGCACTTCA |
| Human-NSD1-Forward | AAGGACAGCCCTTTCGGATG |
| Human-NSD1-Reverse | ACCATGGGCGTCTCTTGAAT |
| Human-NSD2-Forward | CAGGTGTCCTCCACCTCAGT |
| Human-NSD2-Reverse | AGGATTTCTGGTGCCTGCTT |
| Human-CD147-Forward | ACTCCTCACCTGCTCCTTGA |
| Human-CD147-Reverse | GCCTCCATGTTCAGGTTCTC |
| Human-CCL5-Forward | GCTGCTTTGCCTACATTGCC |
| Human-CCL5-Reverse | TCGGGTGACAAAGACGACTG |
